# Atomistic picture of conformational exchange in a T4 lysozyme cavity mutant: an experiment-guided molecular dynamics study†
†Electronic supplementary information (ESI) available: Additional figures, a table and details regarding the NMR experiments, MD simulations and MSM analysis in a single pdf file, a pdb file containing the coordinates of the MD derived B state structure, a single pdb file with five structures from each of the nineteen states and two movies showing the molecule transitioning from the E to B conformers in trajectories 1 and 2. See DOI: 10.1039/c5sc03886c


**DOI:** 10.1039/c5sc03886c

**Published:** 2016-01-07

**Authors:** Pramodh Vallurupalli, Nilmadhab Chakrabarti, Régis Pomès, Lewis E. Kay

**Affiliations:** a TIFR Centre for Interdisciplinary Sciences , 21 Brundavan Colony, Narsingi , Hyderabad 500075 , India . Email: pramodh@tifrh.res.in; b Molecular Structure and Function , Hospital for Sick Children , Toronto , ON , Canada M5G 1X8; c Department of Biochemistry , University of Toronto , Toronto , ON , Canada M5S 1A8; d Departments of Molecular Genetics and Chemistry , University of Toronto , Toronto , ON M5S 1A8 , Canada . Email: kay@pound.med.utoronto.ca

## Abstract

Despite the importance of dynamics to protein function there is little information about the states that are formed as the protein explores its conformational landscape or about the mechanism by which transitions between the different states occur. Here we used a combined NMR spin relaxation and unbiased molecular dynamics (MD) approach to investigate the exchange process by which a cavity in an L99A mutant of T4 lysozyme (T4L 99A) interconverts between an empty and occupied form that involves repositioning of an aromatic residue, Phe114. Although structures of the end-states of the exchange process are available, insight into the mechanism by which the transition takes place cannot be obtained from experiment and the timescales involved are too slow to address using brute force MD. Using spin relaxation NMR methods, we have identified a triple-mutant of T4L that undergoes the same exchange process as T4L L99A but where the minor state lifetime has decreased significantly so that the spontaneous conformational transition to the major state can be studied using all-atom MD simulations. The simulation trajectories were analyzed using Markov state models and the energy landscape so obtained is in good agreement with expectations based on NMR studies. Notably there is no large-scale perturbation of the structure during the transition, multiple intermediates are formed between the two similar exchanging conformations and the free energy barrier between these two well-folded, compact forms is small (6*k*_B_*T*), only slightly larger than for processes considered to be barrierless.

## Abstract 2

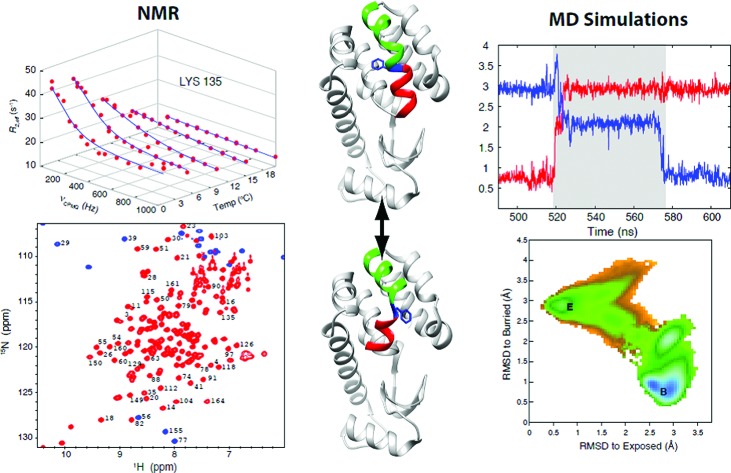
Relaxation-dispersion NMR techniques and molecular dynamics simulations have been used to understand how a cavity mutant of T4 lysozyme interconverts between two compact conformations.

## Introduction

Proteins can adopt a wide range of different conformations in solution.^1^–^3^ Some of these are important for function, affecting processes such as ligand binding, enzyme catalysis, molecular recognition and signaling, for example.^4^–^9^ Intermediate conformations can also be populated during protein folding, influencing the kinetics of formation of the native state as well as, potentially, the formation of aggregates that ultimately lead to disease.^4^,^10^,^11^ The populations of these conformational states depend on their free energies while their lifetimes are a function of a number of factors, including the heights of the free energy barriers that separate them from other states.^12^,^13^ Many of the sampled conformers are sparsely populated and transiently formed, so that they cannot be studied by most biophysical techniques, despite the obvious importance to do so. Moreover, even less is known about the mechanisms by which proteins undergo transitions between the myriad of states that comprise their energy landscapes.

**Fig. 1:**
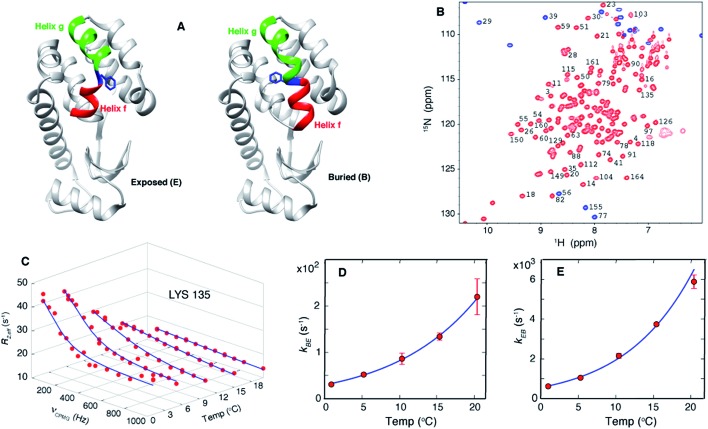
Exchange between E and B conformers of T4L L99A can be quantified using CPMG RD NMR. (A) E and B structures of T4L L99A, as established from X-ray^37^,^40^ and CPMG RD NMR^33^ studies. Helices f (residues 107–113) and g (115–123) are colored in red and green respectively, with Phe 114 shown in blue. (B) ^15^N-^1^H HSQC spectrum^98^ of T4L L99A, G113A, R119P recorded at 35 °C, 11.7 T, with assignments of selected peaks. Peaks aliased in the ^15^N dimension are shown in blue. (C) Amide ^15^N CPMG RD profiles of Lys135 recorded at 1, 5.3, 10.4, 15.4 and 20.4 °C, 11.7 T. Red dots are the measured *R*_2,eff_ rates and the blue line is from a global best-fit to the experimental data. (D, E) Arrhenius plots of *k*_BE_ and *k*_EB_ obtained from analysis of the CPMG data. Experimental rates are shown as red circles and the best-fit Arrhenius curves are in blue (see ESI,† Arrhenius analysis of the temperature-dependent rate constants *k*_BE_ and *k*_EB_).

In the past decade there have been advances in experimental methods that bring into focus transiently-populated protein states and examples of their characterization have emerged using a number of different approaches including single-molecule^14^ and solution Nuclear Magnetic Resonance (NMR) techniques.^15^,^16^ Among the NMR methods, the Carr–Purcell–Meiboom–Gill (CPMG) relaxation dispersion (RD) experiment can be used to detect minor conformers that are populated to as low as 0.5% and have lifetimes between 0.5 and 5 milliseconds (ms).^15^,^16^ The CPMG experiment has been used to study conformational exchange processes involved in enzyme mechanisms,^17^–^19^ protein folding and misfolding^10^,^20^,^21^ and ligand binding.^18^,^22^–^24^ Recent advances in the CPMG RD methodology^25^–^30^ have made it possible to obtain atomic resolution models of some of these transiently-populated states,^31^,^32^ including structures of folding intermediates of the Fys SH3 (ref. 10) and FF domains^21^ as well as structural models of a minor conformer of T4 lysozyme L99A (T4L L99A) (Fig. 1A).^33^

Over the past several decades T4 lysozyme (T4L) has emerged as a model system to understand aspects of protein structure and stability.^34^–^39^ Seminal studies of the effects of mutations on this enzyme have significantly influenced our understanding of the physical chemical properties of proteins in general.^34^ A particularly interesting mutant is T4L L99A, where Leu at position 99 is replaced by Ala.^37^ Substitution of Leu with a smaller residue does not affect the structure of the protein but it does create a 150 Å^3^ cavity in the core of the C-terminal domain, a clear indication of the fact that proteins can be plastic and highly tolerant to even non-conservative mutations.^37^,^40^ Notably, T4L L99A binds hydrophobic molecules such as benzene in the cavity without altering its structure^37^,^40^ and the binding kinetics are rapid,^35^ despite the lack of a clear pathway for substrate entry. CPMG RD NMR studies established that T4L L99A visits a second conformation with a fractional population of approximately 3% and a lifetime on the order of 1 ms at 25 °C (ref. 41) and the major conformer in solution corresponds to that determined by X-ray diffraction.^33^,^41^ Notably, the CPMG RD NMR-derived structure^33^ of the minor conformer is compact and well folded and is very similar in many respects to the major state that has been characterized by X-ray studies.^37^,^40^ The main difference lies in the conformation of Phe114, a residue between helices f and g in the major T4L L99A conformer (Fig. 1A, exposed), where *ψ* changes from ∼+55° in the ground state structure to a helical value of ∼ -50° in the sparse state, along with a concomitant merging of helices f and g to form a long helix in the minor conformer (Fig. 1A, buried).^33^ This change in *ψ* reorients the solvent-exposed Phe114 sidechain in the major state such that it occupies the cavity created by the L99A mutation, becoming buried in the core of the C-terminal domain in the minor conformer (Fig. 1A). In what follows we will refer to the buried and exposed states as B and E, respectively.

Despite the fact that the endpoints of the structural transition of Phe114 are well defined, there is little information on the path(s) by which this transition occurs. Questions relating to the possibility of local unfolding events that allow Phe114 to enter the protein core, the size of the activation free energy barrier, whether there are intermediates that are populated as the protein transitions between states and the pathways taken during the transition, are difficult to address by experiment. Moreover, the atoms or groups of atoms in a protein, such as the Phe114 sidechain in the case of T4L L99A, continuously collide with objects of similar size such as water and other protein atoms, so that their motion is stochastic in nature and difficult to intuit.^6^,^7^,^42^ However, in favorable situations the questions posed above can be addressed using molecular dynamics (MD) simulations. In a conventional classical MD simulation, Newton's equations of motion are numerically solved to generate a movie that shows how the positions of the atoms in the system evolve with time.^43^–^45^ Here the system consists of the protein surrounded by solvent. The atoms interact with one another according to the parameters of the force field that is used and thus the results of the simulation will only be a useful substitute for experiment if the force field accurately models the energy surface on which the protein moves. Thanks to continued increases in computational power^46^ and improvements in force fields and methodology, MD simulations can be used to obtain insights that are not yet available from experiment and applications to a large number of biochemical processes have emerged.^47^–^50^

Despite the improvements in MD approaches outlined above, the issue of timescale is very real. Protein molecules spend most of their time in stable states so that the transitions between states are rare. For example, at 25 °C the major and minor states of T4L L99A have lifetimes on the order of 30 ms and 1 ms, respectively.^33^,^41^ Starting simulations from the buried, CPMG-derived minor state rather than the major state X-ray structure reduces computational time by a factor of 30. However, it still remains impractical to carry out several milliseconds of MD simulation that would be required to get the proper statistics so that the analyzed trajectories can be interpreted with confidence. Different methods have been proposed to overcome these limitations, including importance-sampling techniques such as umbrella sampling^51^ or transition path sampling^52^ and strategies like metadynamics^53^ that use bias to facilitate a more complete exploration of the rugged free energy surface. Because we wished to avoid using any sampling bias to facilitate B to E interconversions we searched instead for suitable T4L mutants that might speed up the exchange process to the point where direct MD simulations would be possible. A candidate emerges in the form of a triple mutant of T4L, referred to subsequently as T4Ltm, into which the L99A, G113A and R119P mutations have been introduced.^33^ The populations of the T4Ltm major and minor states have been shown previously to undergo an inversion relative to T4L L99A whereby B and E now become the major and minor states, respectively,^33^ and importantly for the present work the E state has a lifetime of approximately 25 μs at 37 °C, so that direct MD simulations to study the B/E transition become viable. We show that the CHARMM27 force field^54^,^55^ models the underlying free energy surface well and that unbiased MD simulations starting from the E state are able to transition to the B conformer. Despite the structural similarity between B and E conformers, the transition between the two involves intermediate states, with different trajectories sampling different intermediates. Interestingly, the activation barrier for the E to B transition is small, ∼6*k*_B_*T*, establishing that proteins can interconvert between two folded and compact forms without the need to surmount a large free energy barrier. More generally, our results establish the high degree of complementarity between MD and CPMG RD NMR and show that in combination both methods can provide a detailed description of conformational transitions in proteins.

## Materials and methods

### NMR sample

A 1.5 mM [U-^15^N, ^13^C] T4L L99A, G113A, R119P sample dissolved in 50 mM sodium phosphate, 25 mM NaCl, 2 mM EDTA, 2 mM NaN_3_ pH 5.5, 10% D_2_O buffer was prepared as described previously^56^ and used for all NMR experiments.

### NMR spectroscopy

^15^N CPMG RD data sets were recorded at 1, 5.3, 10.4, 15.4 and 20.4 °C using an ^15^N TROSY CT-CPMG pulse scheme.^27^,^57^ Data sets were recorded at field strengths of 11.7 T (all temperatures) and at 18.8 T (1, 15.4, 20.4 °C). ^1^H CPMG data sets^58^ were also recorded at 11.7 T (10.4 °C) and at 18.8 T (15.4, 20.4 °C) to supplement the ^15^N data. For the ^15^N experiments the constant time relaxation delay, *T*_CPMG_, was set to 20 ms (1 °C) or 30 ms (5.3, 10.4 °C) and 24 ms (15.4 and 20.4 °C), while a value of 16 ms was used for recording the ^1^H CPMG data at 10.4 °C and 18 ms at 15.4 and 20.4 °C. Dispersion profiles comprised ∼15 different *ν*_CPMG_ frequencies, recorded in an interleaved manner, with values ranging from the minimum possible value (1/*T*_CPMG_) to a maximum of 1000 Hz in the case of the ^15^N experiments and 2000 Hz for the ^1^H experiment. Errors were estimated on the basis of repeat measurements at two or three different *ν*_CPMG_ frequencies. The temperature was measured by using a thermocouple inserted into an NMR tube that was placed in the magnet. Each CPMG dataset required approximately 20 hours of measurement time. All experiments were performed on Varian Inova spectrometers equipped with room temperature triple resonance probes. (See ESI,† NMR data processing and analysis for additional details).

### MD simulations

All MD simulations were performed with the GROMACS 4.5.5 package,^59^ as described in detail in the ESI.† MSM analysis of the MD data was carried out using a combination of the MSMBuilder^60^ and EMMA^61^ software packages, supplemented with in-house written scripts. The Chimera^62^ and VMD^63^ programs were used to view the structures and trajectories.

## Results

### CPMG NMR shows that the T4Ltm minor state accesses the major conformation on a timescale that can be simulated

CPMG RD NMR spectroscopy has been used to study the kinetics and thermodynamics of the interconversion of T4Ltm between states B and E. In the CPMG experiments^64^,^65^ used here, transverse magnetization is allowed to evolve for a constant time *T*_CPMG_ during which chemical shift refocusing pulses are applied with a frequency of *ν*_CPMG_. For systems undergoing conformational exchange between different states the chemical shifts of the NMR active reporter nuclei fluctuate stochastically with time. When these fluctuations occur on the millisecond timescale the chemical shift evolution of the reporters can be partially refocused by the application of refocusing pulses during *T*_CPMG_. This leads to signal intensities that, in general, are lower than what would be observed in the absence of exchange and these intensities can be recast in terms of site specific relaxation rates, *R*_2,eff_. The resulting profile of *R*_2,eff_ as a function of *ν*_CPMG_, the so called RD profile, is fit to the appropriate model of chemical exchange to extract the kinetics and thermodynamics of the underlying exchange process as well as the chemical shifts of the minor and most often invisible state(s). As chemical shifts are exquisitely sensitive to structure, CPMG experiments can be used to detect and characterize conformers whose structures are only subtly different from one another^31^,^33^ and that are difficult to distinguish by other techniques.^20^

As discussed above, we have previously established that introduction of the G113A, R119P mutations into the T4L L99A background inverts the populations of the ground and excited states.^33^ RD CPMG measurements on samples of T4L L99A and T4Ltm are expected, therefore, to report on the same exchange process and this has been demonstrated experimentally.^33^Fig. 1B shows a well resolved ^15^N-^1^H HSQC spectrum^33^,^66^ of T4Ltm. The high spectral quality facilitates recording CPMG RD data sets that can then be analyzed to obtain accurate exchange kinetics and thermodynamics as a function of temperature (Table 1, see ESI†). Fig. 1C shows RD profiles for Lys135 obtained between 1 and 20.4 °C. Dispersion profiles from all residues at each temperature (typically between 8 and 21 residues, see ESI† Materials and methods) were fit together to a two-state exchange model (Fig. S1†) that we have previously shown to be appropriate for the B–E interconversion.^33^,^66^ The temperature dependent exchange rates *k*_BE_ and *k*_EB_ obtained from the two state analysis are plotted in Fig. 1D and E. These rates were analyzed using the Arrhenius equation to extract Δ*H* and Δ*S* values for the exchange reaction and the enthalpy of activation, ΔH* (Fig. S1†). The minor state of T4Ltm (state E) has a relatively short lifetime of approximately 160 μs at 20.4 °C that decreases steeply with increasing temperature (Fig. 1D and E, Fig. S2,† Table 1). This comes about from two effects. First, the large ΔH* (65 kJ mol^–1^, Fig. S1,† Table 2) that increases exchange rates and second because the E to B reaction is endothermic (Table 2). Thus, the population of the minor E state goes down with increasing temperature (Fig. S2B†) accelerating the rate of E to B conversion still further. This combined effect of decreasing both the lifetime and the population of E as the temperature is raised is reflected in Fig. 1C where the size of the RD profiles decreases with increasing temperature. Important for the work described below, the lifetimes of the E state are estimated to be ∼25 μs at 37 °C and ∼7 μs at 50 °C by Arrhenius extrapolation of the rates obtained from ^15^N and ^1^H RD CPMG experiments (Fig. 1C–E, Fig. S2,† Table 1). This suggests that it should be possible to observe a number of transitions to state B by carrying out MD simulations (both at 37 °C and 50 °C) that start from state E. Simulations were not performed at higher temperatures than 50 °C because we have observed that, while T4Ltm is stable at 37 °C, samples aggregate over approximately 30 minutes at 50 °C. Most of the discussion in the present work focuses on MD results from simulations at 37 °C, although very similar conclusions are obtained from the higher temperature MD trajectories as well.

**Table 1:** Kinetic and thermodynamic parameters for the B–E exchange process obtained from an analysis of temperature dependent CPMG RD NMR experiments, a 19 state MSM and direct MD simulations^*a*^

Temp (°C)	*k*_ex_ (s^–1^)	*p*_E_ (%)	Source
1.0	649 ± 50	4.7 ± 0.2	CPMG RD NMR
5.3	1099 ± 57	4.7 ± 0.2	CPMG RD NMR
10.4	2240 ± 122	3.8 ± 0.5	CPMG RD NMR
15.4	3871 ± 79	3.5 ± 0.2	CPMG RD NMR
20.4	6100 ± 350	3.6 ± 0.6	CPMG RD NMR
37	3.9 ± 0.3 × 10^4^	2.3 ± 0.3	Arrhenius extrapolation of CPMG RD NMR derived rates
37	3.3 ± 0.4 × 10^5^	1.4 ± 1	19 state MSM (2 state analysis)
50	1.3 ± 0.3 × 10^5^	1.9 ± 0.3	Arrhenius extrapolation of CPMG RD NMR derived rates
50	6.6 ± 0.3 × 10^5^	0.6 ± 0.3	19 state MSM (2 state analysis)

Transition path time (ns)			
	Average value	Range	Source
37	60.5	2.4 to 231.2	MD simulations
50	39.6	1.3 to 141.6	MD simulations

^*a*^To estimate two state exchange parameters from the 19 state MSM 1/*k*_ex_ was taken to be the slowest implied timescale as it corresponds to the E–B interconversion (Fig. 3) and states E and B were defined in terms of the populations of states 0 (E) and 15–18 (B), respectively. The fractional population of state E, *p*_E_, was then calculated as the population of state 0/(sum of populations of states 0 and 15–18). The value of *p*_B_ = 1 – *p*_E_.

**Table 2:** Thermodynamic parameters for the two state model obtained using a combination of Arrhenius analysis of the experimental rates along with TPTs obtained from the MD simulations. All parameters are defined with the major state B as the reference, that is Δ*X* = *X*_E_ – *X*_B_ (Fig. S1)

Parameter	Value	Source
Δ*H*	–15 ± 3 kJ mol^–1^	Arrhenius fits to CPMG NMR derived rates
Δ*S*	–79 ± 11 J mol^–1^ K^–1^	Arrhenius fits to CPMG NMR derived rates
Δ*G*	9.6 ± 0.3 kJ mol^–1^ at 37 °C	Arrhenius fits to CPMG NMR derived rates
	10.6 ± 0.4 kJ mol^–1^ at 50 °C	Arrhenius fits to CPMG NMR derived rates
ΔH*	65 ± 4 kJ mol^–1^	Arrhenius fits to CPMG NMR derived rates
ΔS*	128 ± 14 J mol^–1^ K^–1^ at 37 °C	Arrhenius fits to CPMG NMR derived rates and TPTs from the long MD simulations for both 37 and 50 °C data
	125 ± 13 J mol^–1^ K^–1^ at 50 °C	
ΔG*	25.3 ± 1.3 kJ mol^–1^ at 37 °C	Arrhenius fits to CPMG NMR derived rates and TPTs from the long MD simulations for both 37 and 50 °C data
	24.7 ± 1.1 kJ mol^–1^ at 50 °C	

### MD simulations reproduce experimental observations

**Fig. 2:**
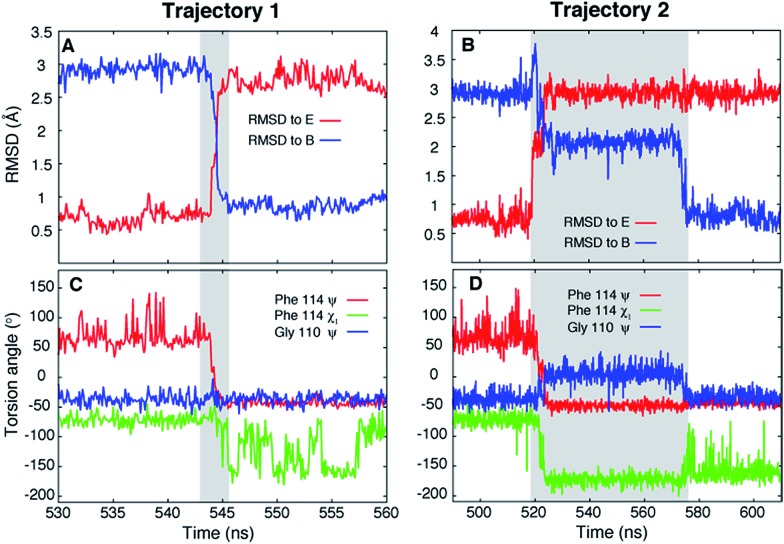
Unbiased MD simulations, 37 °C, capture the T4Ltm E to B transition. (A, B) RMSD to the E (3DMV (ref. 40)) and B (see ESI,† details of the MD simulations) states as a function of time for two trajectories that capture the E to B transition. The trajectories start in the E state and finish in the B state. The N, Cα, carbonyl C and O atoms of residues 100 to 120 and all heavy atoms in Phe114 and Leu133 were used to calculate RMSD values. (C, D) Variation of Phe114 *ψ*, *χ*_1_ and Gly110 *ψ* torsion angles as a function of time. The intervals during which a molecule transitions from E to B are shown in grey. Movies showing the E to B transitions for trajectories 1 and 2 are available in the ESI.†

Ten spontaneous transitions from E to B were observed during eighty ∼1 μs MD simulations at 37 °C and seventeen transitions occurred in forty ∼1 μs simulations at 50 °C. All of the simulations were run starting from the E conformation of T4Ltm, thus exploiting the short lifetime of the E state to increase the likelihood of transitions. Two of the transitions observed in simulations at 37 °C are shown in Fig. 2 (movies showing the E to B transition for these two trajectories are available in the ESI†). In both cases the protein explores conformations close to the starting E structure (red curves in Fig. 2A and B), corresponding to the crystal structure of T4 L99A (RMSD < 0.8 Å)^40^ which is the ground state of the L99A mutant^33^,^41^ and the minor state for T4Ltm. The protein then transitions to B either rapidly (3 ns, Fig. 2A) or somewhat slower (60 ns, Fig. 2B) and then remains stably in state B. It is particularly noteworthy that the MD ensemble corresponding to state B (ground state of T4Ltm) is similar to the CPMG RD-derived structure of the sparsely populated state of L99A where Phe114 is bound inside the cavity (Fig. S3, see ESI,† details of the MD simulations). Analysis of the distribution of the Phe114 *χ*_1_ angle indicates an average value of ∼–60° in the E state (Fig. 2C and D) that transitions predominantly to a trans value (180°) in the B state (Fig. 2C and D) in agreement with experiment.^33^,^40^ Thus, the CHARMM27 force field used here places the minima in the free energy surface at positions that are in agreement with those obtained from NMR experiments and further is able to capture subtle aspects of sidechain conformation correctly.

To test whether the force field reproduces the experimentally predicted populations and exchange rates, several short (100 ns) simulations were run starting from molecules that were transitioning from E to B (see ESI,† details of the MD simulations). As expected, at the end of the simulations conformations in both E and B states were observed. The resulting trajectories were subsequently analyzed using a Markov State Model (MSM) approach which has proven to be powerful in the interpretation of large MD data sets and the complex transitions that are often associated with conformational changes in biomolecules.^67^–^72^ The method is appealing in that it does not require knowledge of the appropriate reaction coordinate that describes the transition, which is often very hard to estimate. In addition to providing a simple intuitive picture of the underlying exchange processes the MSM approach generates quantitative estimates of rates and populations.^71^,^72^ In the MSM procedure the underlying dynamics are modeled as memoryless transitions between different microstates in phase space. In our study the structures from approximately 140 μs of MD trajectories were clustered into 200 microstates based on RMSD and dihedral angle criteria (see ESI, MSM analysis of the MD trajectories), although other clustering metrics, including varying the number of microstates (Fig. S4E and F†), gave very similar results. In this approach each time point in each of the trajectories corresponds to a microstate, giving rise to a set of microstate *vs.* time trajectories. These annotated trajectories are then used to construct a transition matrix, *T*(*τ*), where *T*_*i*,*j*_(*τ*) is the probability of transitioning from microstate *i* to microstate *j* after a lag-time *τ*.^71^,^73^ The evolution of the populations of the microstates over a time *nτ* can then be calculated according to the relation, *P*(*nτ*) = *P*(0)*T*(*τ*)^*n*^, where *P*(*t*) is a vector with each element *j* corresponding to the population of the *j*th microstate at time *t* and *n* is an integer. The eigenvectors of the matrix *T*(*τ*) are called the transition modes that describe transitions between the different microstates and the eigenvalues *λ* of *T*(*τ*) are related to the characteristic time scales (implied time scales) of these transitions according to 
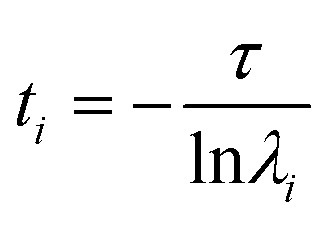
, where *λ*_*i*_ is the *i*th eigenvalue. The first eigenvector gives the equilibrium populations of the system and its associated time scale, *t*_1_, is infinity (*i.e.*, the equilibrium distribution of the system does not evolve). To obtain an intuitive picture of the dynamics, the many microstates are typically lumped into a smaller number of macrostates that can then be interpreted more easily. In our analyses the 200 microstates were clustered into 19 macrostates (states 0 to 18) (see ESI, MSM analysis of the MD trajectories, Fig. S4 and S5†). All of the structures in a given macrostate are very similar (Fig. S6†) so that the underlying dynamics can be understood in terms of transitions between this small number of conformations. Transition path theory^74^ was then used to obtain the flux of the various pathways connecting E to B along with commitor values of each of the macrostates that provides a measure of the ‘closeness’ of that macrostate to E and B. As used here, a commitor value of less than 0.5 (50%) indicates that trajectories originating from this state are more likely to visit E before B and a commitor greater than 0.5 (50%) implies the opposite. Transition states have commitor values of approximately 0.5 (50%).^75^,^76^ Based on the RMSD values to the B and E structures and commitor values from a preliminary analysis, macrostate 0 was defined as the E state and states 15–18 as the B state. Here the states have been arranged according to increasing commitor values.

**Fig. 3:**
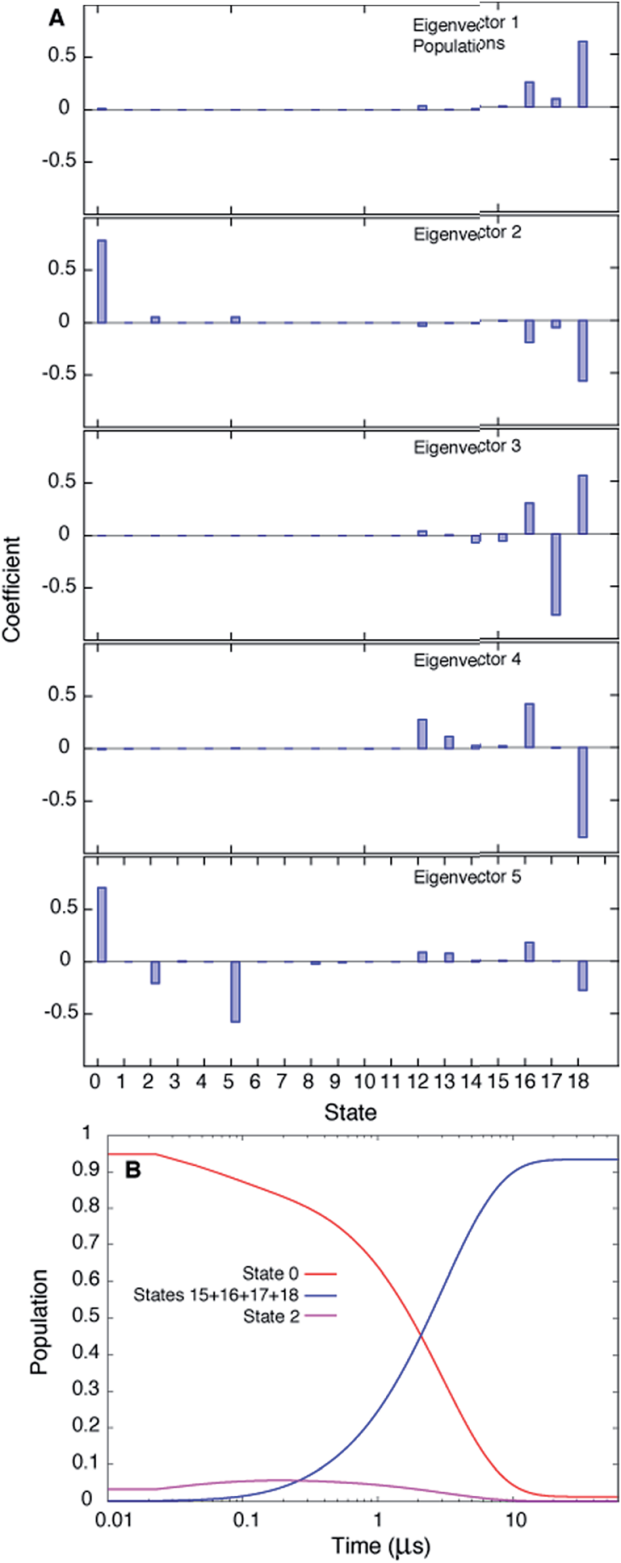
The slowest process detected in the MSM analysis of the MD simulations of T4Ltm, 37 °C, corresponds to the E to B interconversion. (A) The first five eigenvectors of the 19-state MSM. The states are ordered according to their committor (Table S1†), that is the probability of reaching B before E. States 8 and 9 are transition states with committor values ∼0.5. The first eigenvector gives the steady state populations of each of the 19 macrostates and the next four correspond to the four slowest implied timescales. The second mode (eigenvector 2) reports the exchange between B and E states of T4Ltm that can be seen from the fact that the state space is partitioned into states 0 and 16–18 that in turn correspond to E and B, respectively. (B) Time evolution of the population of state 0 and the sum of the populations of states 15–18, starting from an initial population of state 0 of 1, while all the other states are not populated. The system is evolved using the transition matrix at *τ* = 22.5 ns according to the relation *P*(*nτ*) = *P*(0)*T*(*τ*)^*n*^, where *P*(0) is a population vector with the first element 1 and all other elements 0. The population of state 0 decreases along with a concomitant increase in the sum of the populations of states 15–18, with a time constant of ∼3 μs that corresponds to the slowest process observed. The population of state 2 transiently goes up and comes down.

**Fig. 4:**
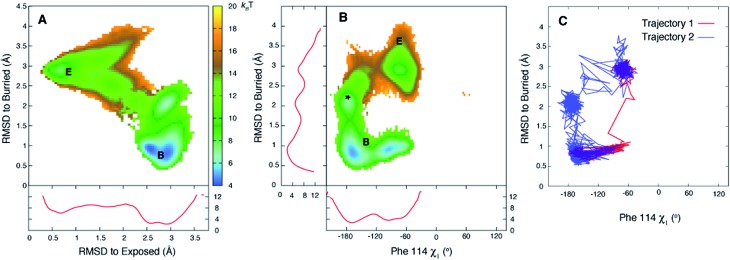
The free energy surface is rugged over the small length scale that separates states E and B. (A, B) Free energy surfaces calculated at 37 °C using the 19-state MSM. Three minima are observed for state B (middle panel) corresponding to the *χ*_1_ torsion angle of Phe114 populating different rotamer states, with the trans conformation the predominant one (see Fig. S7†). Notably minima other than for B and E are observed as well (*). Red curves in panels (A) and (B) correspond to 1D PMF surfaces, corresponding to projections of 2D energy surfaces onto a single axis. (C) The E to B transition can occur *via* different pathways as illustrated for trajectories 1 and 2 of Fig. 2, showing that the molecule in trajectory 2 becomes trapped in the minimum labeled by * in (B). Atomic coordinates for the reference B state structure are available in the ESI.†

Fig. 3A plots the first 5 eigenvectors of the 19 × 19 transition matrix (37 °C) with the first eigenvector giving the equilibrium populations of each of the macrostates. As expected the populations are highly skewed towards B with more than 93% of the total conformers populating B at equilibrium. The second eigenvector, with the slowest implied timescale *t*_2_ (*t*_1_ is infinity as mentioned above), reports on the interconversion between states B and E, as reflected by the opposite signs of the elements of the vector in the regions of state space corresponding to B and E. The time dependencies of the populations of states B (macrostates 15–18) and E (macrostate 0) have been calculated from the transition matrix as shown in Fig. 3B and are decidedly two-state. Indeed, equilibrium populations given by eigenvector 1 (1.3% for state E), as described above, and the lifetime of the E state obtained from the slowest implied timescale (3 μs, Fig. S4,† red) are in reasonably good agreement with results from the (two-state) analysis of the experimental NMR data extrapolated to 37 °C (2.3% and 26 μs), Table 1. Free energy surfaces calculated using the MSMs clearly show minima at states B and E (Fig. 4A and B, S7†). Notably, however, the landscape is more complex than two state, as can be seen in Fig. 4B. The rugged landscape can explain the different trajectory profiles in Fig. 2A and B since trajectory 2 (Fig. 2B) involves formation of stabilizing intermediates that slow down the E to B transition, Fig. 4C, compared to trajectory 1, in which the exchange is direct.

**Fig. 5:**
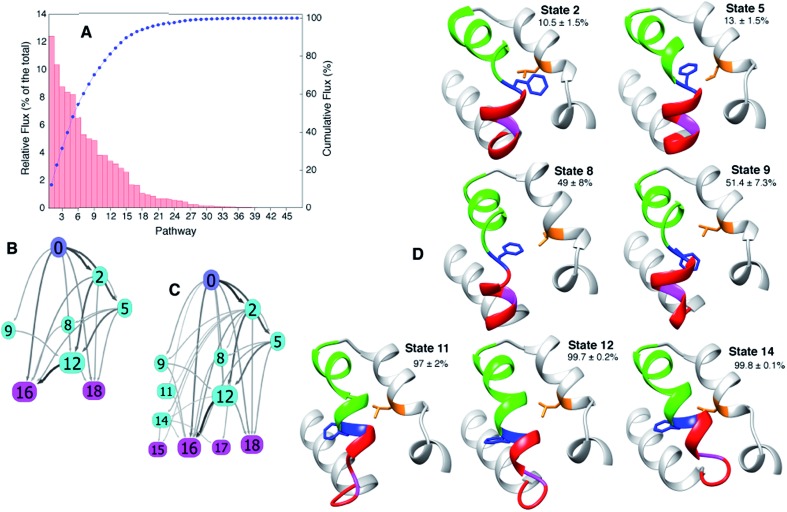
The paths connecting E to B involve different intermediates. (A) Relative contribution of each of the 47 pathways calculated from the MSM to the total flux, 37 °C. (B, C) The most important 11 and 22 pathways are shown in (B and C), respectively, with the arrows shaded according to the flux between each pair of states. (D) Dominant intermediates in the E to B interconversion reaction. Residues 94 to 140 of T4Ltm are shown in ribbon format: helix f in red, helix g in green, Gly 110 in magenta, Phe 114 in blue and Leu 133 in orange. Sidechains of Phe 114 and Leu 133 are indicated and the committor values for the E to B transition listed as percentages. Atomic coordinates of five randomly chosen structures from each of the 19 states are available in the ESI.†

In addition to obtaining rates and populations it is also possible to deduce both the pathways that connect E and B and the relative flux from each pathway^68^,^74^,^77^ using the transition matrix *T* and transition path theory.^68^,^74^ Applying transition path theory to the 19-state MSM used here establishes 47 paths connecting E and B, each involving different intermediates. These do not contribute equally to the flux, with 11 paths accounting for 75% of the total flux (Fig. 5A–C).

### Similar trajectories for T4Ltm and T4L L99A

**Fig. 6:**
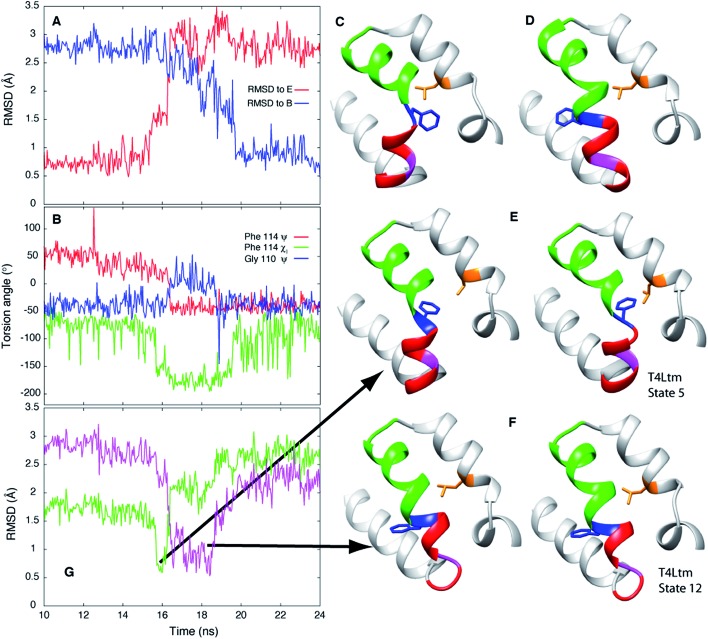
E to B transitions for T4Ltm and T4L L99A proceed *via* similar pathways. (A) RMSD to E and B states during the course of a high temperature MD trajectory (167 °C) starting from the T4L L99A X-ray structure (3DMV^40^). The molecule starts in state E and transitions to B. (B) Variation of the Phe114 *ψ*, *χ*_1_ and Gly110 *ψ* torsion angles as a function of time. (C, D) Structures of the T4L L99A E (C) and B (D) states obtained from the simulations. (E, F) Key intermediates showing the formation of non-native interactions (E) or distortion of the N-terminus of helix F (F) during the T4L L99A E to B transition (left hand sides of panels) are very similar to those generated during the T4Ltm trajectory (right hand sides). The intermediates in panels (E) and (F) are similar to the T4Ltm states 5 (E) and 12 (F), with Gly 110 in panel (F) (magenta) adopting a non-helical conformation. (G) RMSD values to intermediates in panels (E and F), shown in green and pink, respectively. Notably, the MD simulations of T4L L99A show that the intermediate corresponding to state 5 in the T4Ltm E to B transition is formed before the intermediate that is structurally similar to state 12, as observed in the T4Ltm trajectory. The N, Cα, carbonyl C and O atoms of residues 100 to 120 and all heavy atoms in Phe114 and Leu133 were used to calculate RMSD values. Residues 94 to 140 are shown in ribbon format: helix f in red, helix g in green, Gly 110 in magenta, Phe 114 in blue and Leu 133 in orange with the sidechains of Phe 114 and Leu 133 indicated.

In order to establish if the pathways by which E to B interconvert are similar for T4Ltm and T4L L99A we have performed high temperature MD simulations (167 °C) starting from the crystal structure (3DMV^40^) of T4L L99A. An example of a trajectory that makes the E and B transition is shown in Fig. 6. To test if the free energy surface for T4L L99A is similarly rugged as for T4Ltm, a series of 500 MD simulations were performed at 37 °C (each of duration 100 ns), starting with structures obtained from the high temperature simulation over the intervals where transitions from E to B occurred. The 500 endpoint structures that were obtained were subsequently analyzed. We found that both E and B state structures were formed, along with conformers that were similar to those populated by T4Ltm during the E to B transition (Fig. 6). Notably, the non-native interactions that are observed in the case of the T4Ltm intermediates can also be seen during the transition of T4L L99A, as discussed below and illustrated in Fig. 6. Thus, despite the introduction of two additional mutations in generating T4Ltm from T4L L99A, leading to an inversion of the populations of states B and E, there are similarities in the free energy surfaces of both proteins and consequently in the interconversion pathways (see below).

## Discussion

Protein conformational transitions are often of critical importance for biomolecular function, yet in most cases our understanding of how such processes occur is extremely limited. This is in large part a reflection of the fact that experimental methods tend to focus on highly populated long-lived conformations, while sparsely populated and transiently formed states remain invisible. Only recently have atomic resolution models for some of these invisible states become available through experiment^10^,^21^,^31^,^33^ and even in these cases little is known about how the transition between these states occurs. Herein we address this problem for a cavity mutant of T4 lysozyme interconverting between a pair of states whereby an exposed aromatic residue (Phe114) becomes buried inside the cavity. Using a combined mutagenesis, NMR and MD approach we provide an atomic-level description of the transition that is not possible from experiment alone. Critical to the success of our strategy has been the identification of a triple mutant of T4L with a much shorter lifetime of the transient state, corresponding to the empty cavity form. CPMG RD NMR studies establish that the lifetime of this state in T4Ltm is on the order of 25 μs at 37 °C (*via* extrapolation, Table 1), so that direct MD simulations become viable. Moreover, experimental RD data unequivocally show that the endpoints of the interconversion processes of both T4Ltm and L99A are identical (although reversed). A second important advance has been the development of force fields that provide an avenue for carrying out such detailed studies. We have established that the CHARMM27 force field used to simulate the E to B transition of T4Ltm generates minima in the free energy surface at positions that are in agreement with those from NMR experiments, thus providing confidence that the MD trajectories will provide robust details about the E/B exchange dynamics that cannot be obtained from experiment.

In addition to being able to simulate the E to B T4Ltm transition correctly, the calculated populations and kinetics, assuming a two state interchange, are in reasonably good agreement with extrapolations of the experimentally measured values. Notably, the population is in better agreement (1.3 ± 1.1% *vs.* 2.3 ± 0.3%, Table 1, 37 °C) than the exchange rate (3.3 ± 0.4 × 10^5^ s^–1^*vs.* 3.9 ± 0.3 × 10^4^ s^–1^, 37 °C). Faster calculated kinetics from the MD data may result from a number of factors including that (i) force fields have errors on the order of *k*_B_*T*, (ii) the TIP3P water model used in the simulations has a diffusion constant that is higher than the experimentally measured value^78^ and (iii) incorrect partitioning of structures into different states leads to an overestimate of rates. It must also be kept in mind that the NMR RD experiments could not be performed at temperatures higher than 20 °C because exchange rates become too rapid, so the values are based on extrapolations assuming an Arrhenius model that may not be fully valid.

Of the 27 trajectories (10 at 37 °C and 17 at 50 °C) for which E converts to B it is noteworthy that there are no large-scale structural rearrangements during the transition. As shown in Fig. 2C and D the E to B interconversion is accompanied by a change in the Phe114 *ψ* torsion angle from +55° to –50°. This change occurs in only one direction, *via ψ* = 0° and not *ψ* = 180°, that corresponds to the shorter trajectory so that the sidechain enters into the page in Fig. 1A to reach the cavity. As described above, molecules can transition from E to B either rapidly, in a few nanoseconds ∼3 ns (Fig. 2A and C, between 543 ns and 546 ns), or can take several tens of ns (Fig. 2B and D between 518 ns and 576 ns). Theory predicts that barrier crossing events are very fast even in the high-friction, overdamped regime.^79^ To understand the underlying mechanism for slower than expected interconversion rates in some cases we calculated the underlying free energy surface. The free energy landscape is rugged (Fig. 4A and B) with minima at positions between B and E (Fig. 4A and B). Trajectory 1 (Fig. 2A and C) uses a path that avoids the minima (Fig. 4C, red), but the molecule traversing trajectory 2 (Fig. 2B and D) gets trapped in one of the minima (Fig. 4C, blue). Hence barrier crossing events between minima are indeed fast as predicted by theory but long transition times arise when the molecule becomes trapped in local minima along the pathway.

The MSM approach used here allows calculation of the different pathways that connect the E and B states, as well as the intermediates that are formed along the pathways. Forty-seven different paths are obtained by applying transition path theory to the 19 state MSM considered here, with 11 paths accounting for 75% of the observed flux between the end-states (Fig. 5A–C). Representative structures of macrostates that are on the most important pathways are highlighted in Fig. 5D along with their committor values (Table S1†). Insight into why some of the intermediates are stabilized can be obtained by inspecting their structures (Fig. 5D, S6 and S8†). For example, in state 5 (committor ∼ 13%) a non-native contact is formed between Phe114 and Leu133 that is possible because *ψ* ∼ 0° for Phe114, in-between the B and E state values, while the *χ*_1_ is trans. State 9 (committor ∼50%) is a transition state with a similar Phe114 *ψ* value as state 5. Unlike state 5, however, Phe 114 *χ*_1_ = ∼–60° so that no contact is formed with Leu133, leaving the molecule in a conformation that can go either to E or B with equal probability. In State 10 (Fig. S8,† committor ∼ 95%), Phe114 is buried in the cavity but the backbone remains similar to the E state allowing the molecule to escape to E ∼ 5% of the time. States 12 and 14 have committor values very close to 1 but Gly110 moves out of a helical conformation, distorting the N-terminal portion of helix G. Notably, even when the exchange is between two similar structures, as in the present case, there are similarities to processes that evoke much larger structural changes, such as protein folding. For example, in both folding and the T4L transition, intermediates have been characterized that serve as kinetic traps,^21^,^80^,^81^ along with nonnative interactions that stabilize conformers that are formed along the pathway,^10^ such as between Phe114 and Leu133 in the case of T4Ltm.

The exact pathways and intermediates observed for the E to B interconversion of the triple mutant could, in principle, be somewhat different than those populated in the exchange reaction of T4L L99A. Nevertheless, an MD study of the latter mutant (see ESI†) as discussed above, has established that the critical intermediates involving non-native interactions between Phe 114 and Leu 133 in one case or the distortion of the N-terminus of helix F that affects the conformation of Gly 110 in another case are present in the pathways traversed by the L99A mutant as well (Fig. 6). Moreover, the order of formation of these intermediates is similar in E to B trajectories for both L99A and T4Ltm. Finally, the E to B interconversion for T4L L99A, as for T4Ltm, involves a change in the Phe114 *ψ* torsion angle from +55° to –50° that occurs *via ψ* = 0° and not *ψ* = 180°, a further demonstration of the similarity of both sets of trajectories. The utility of mutants in experimental biophysical studies is, of course, well documented in the literature and can provide insight into complex processes such as protein folding and enzyme catalysis.^99^ It appears that mutants can also be advantageous in computational studies as well, although with the caveat that care must be taken to ensure that similar trajectories are sampled by wild-type and mutant proteins, as was done here.

The temperature-dependent rate constants, *k*_EB_ and *k*_BE_, have been fitted to an Arrhenius model to extract Δ*H*, Δ*S* and ΔH* values for the T4Ltm interconversion, Table 2 (see ESI,† Arrhenius analysis of the temperature dependent rate constants, *k*_BE_ and *k*_EB_). However, the height of the activation free energy barrier, ΔG*, is not available from experiment. It can be estimated using the relation, 
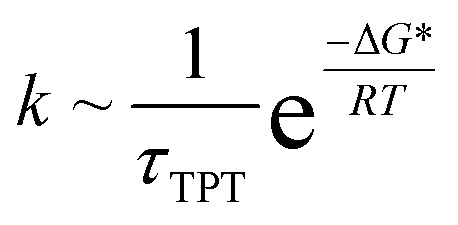
, where *k* is the rate constant and *τ*_TPT_ is the transition path time (TPT), the time it takes for a molecule to transition between two states in the absence of a barrier.^13^ It has been shown that the barrier does not have much of an effect on the calculated TPT^79^,^82^,^83^ so that TPTs measured in the presence of a barrier can be used in the above expression. Although rate constants, which are related to the lifetimes of the interconverting states, are routinely measured, TPTs are extremely challenging to obtain, with the first such values reported only recently.^84^,^85^ The TPTs between states E and B of T4Ltm can be readily estimated from the simulations (see ESI, details of the MD simulations) and they vary from 2.5 to 230 ns at 37 °C (Table 1, Fig. S9†), with an average value of 60.5 ns. Using the extrapolated *k*_BE_ rate based on the experimental measurements (Table 1) the free energy barrier for the B to E transition is calculated to be ∼10*k*_B_*T*, which is significantly different from the measured ΔH* of ∼25*k*_B_*T* (Table 2), arguing against using experimentally-derived activation enthalpies as a good approximation for ΔG*. A value of *T*ΔS* = 15*k*_B_*T* follows directly from ΔG* and ΔH*. The free energy barrier for the reverse reaction, E to B, is calculated in the same way to give ∼6*k*_B_*T*, only a factor of two greater than the 3*k*_B_*T* threshold used to classify reactions as activated.^86^ Using the MD-derived rate constants in the above calculations rather than those from experiment decreases the size of the barrier by ∼2*k*_B_*T* (in both directions), but does not alter the conclusions about the modest barrier size for the interconversion between two compact protein conformations.

Although a small barrier height has been obtained in the case of a designed cavity mutant of T4L considered here, there are reasons to believe that many stochastic processes in naturally-occurring proteins will also involve small activation barriers. For example, small proteins that fold without an activation barrier have been discovered recently.^87^,^88^ Further it may be that molecular machines in the cell function by transitioning between states that involve small barriers as is thought to be the case with the ribosome as it moves along mRNA.^6^ During protein synthesis the ribosome moves from one codon to the next, a distance of ∼20 Å. Simultaneously the A site tRNA moves to the P site and the P site tRNA exits the ribosome through the E site. This process, called translocation, takes place in the presence of the ribosomal factor EF-G and requires GTP hydrolysis. Interestingly the ribosome can translocate in the absence of EF-G,^89^,^90^ suggesting that the barriers involved in this complex process are also not large.^6^ Notably, a large activation enthalpy has been reported (∼36*k*_B_*T* at 25 °C) but this is expected to be countered by a positive *T*ΔS* to make the activation barrier small.^6^ Further, recent NMR relaxation studies have shown that Hoogsteen base pairs in duplex DNA molecules can transiently form^91^ even in the absence of proteins or DNA damage and MD based free energy calculations suggest that barriers can be quite small.^92^ The results obtained here may also have implications for ligand binding to proteins. Proteins such as hemoglobin, myoglobin and T4L L99A, bind ligands in cavities that cannot be accessed from the surface in the major state structure. Our results suggest that ligands can enter the core of the protein without having to surmount large free energy barriers. Finally, it is worth noting that part of the misconception in the literature about barrier heights comes from the fact that most of their estimates have been obtained using Eyring transition state theory,^93^ which assumes that the preexponential factor in the rate equation is 
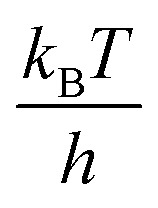
 ∼6 × 10^12^ s^–1^, much larger than the ∼2 × 10^7^ s^–1^ estimated here. The 
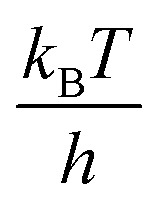
 prefactor is the approximate bond vibration frequency and is not a valid estimate for the relevant frequencies associated with multiple residue conformational changes in proteins.^6^ Indeed, experimentally-measured TPTs for the folding of small proteins range from 2 to 10 μs.^84^ Hence barrier heights estimated using Eyring transition state theory are overestimated by greater than 10*k*_B_*T*. Notably, potential errors in the force fields used in the MD simulations reported here are not expected to change the conclusion of small barrier heights since even if the calculated TPTs are off by an order of magnitude the estimated barrier heights will change by only ±2.3*k*_B_*T*(ln(10) ∼2.3).

## Conclusions

Pioneering NMR studies^94^–^96^ and MD simulations^97^ four decades ago established that aromatic residues in the cores of proteins were not static entities, but rather highly dynamic, undergoing ring flipping motions. An atomic-level description of such motions had to await the development of both improved NMR experiments and computational approaches. Herein, using unbiased molecular dynamics simulations at 37 °C, we have described the dynamics of an exposed aromatic residue in a cavity mutant of T4L that transitions to a buried state inside the cavity. Central to the success of the study was the identification of a mutant of T4L L99A with a short minor-state lifetime corresponding to the exposed conformation so that the transition could be studied using unbiased molecular dynamics simulations. The energy landscape so obtained is in good agreement with expectations from RD CPMG NMR and the populations of states and rates of interconversion from the MD simulations are in reasonable agreement with experimental estimates, providing confidence in the MD results. Notably, there is no significant perturbation to the T4L structure, Fig. 5D, as it transitions between the two conformations, while there are different possible pathways involving intermediates that have been characterized using a MSM approach. Thus, the free energy surface is rugged even on this small length-scale, trapping molecules in local minima that increase TPTs to as long as 200 ns. Interestingly the activation barrier for the E to B transition is 6*k*_B_*T* indicating that two compact protein states do not have to be separated by a large barrier. The success of the combined mutagenesis/RD-NMR/MD approach and the ability of MD to correctly capture the T4L transition observed in solution encourages additional such studies exploiting solution methods to characterize at atomic level sparsely populated states and MD techniques to understand how transitions between such states and the ground state can occur.

## Abbreviations

TROSY: Transverse relaxation optimized spectroscopy
CPMG: Carr–Purcell–Meiboom–Gill
TPT: Transition path time
RD: Relaxation dispersion
MD: Molecular dynamics

## Supplementary Material

Supplementary information

Supplementary information

Supplementary information

Supplementary movie

Supplementary movie
