# Supplementary material for: Atomistic picture of conformational exchange in a T4 lysozyme cavity mutant: an experiment-guided molecular dynamics study
Source: Chem Sci. 2016 Jan 7;7(6):3602–13. doi: 10.1039/c5sc03886c (PMC6008728; doi:10.1039/c5sc03886c)
Supplement: Supplementary file 3 [file SC-007-C5SC03886C-s003.pdf]

Electronic Supplementary Material (ESI) for Chemical Science.
This journal is © The Royal Society of Chemistry 2016

Supporting Information

Atomistic Picture of Conformational Exchange in a T4 Lysozyme Cavity Mutant: An Experiment Guided Molecular Dynamics Study

SI Materials and Methods

NMR data processing and analysis. NMR data were processed using the NMRPipe suite of programs [1] and visualized using SPARKY [2]. Peak intensities were quantified using FuDA (http://pound.med.utoronto.ca/software.html) and analyzed using the in-house written program ChemEx (available from the authors upon request). The effective transverse relaxation rate of amide ^{15}N or ^{1}H spin probes, $R_{2,eff}$, at each CPMG frequency was calculated according to the relation $R_{2,eff} = \frac{-1}{T_{CPMG}} \ln\left(\frac{I}{I_o}\right)$ where I is the peak intensity in the spectrum and I_o is the corresponding intensity in a reference spectrum recorded without the constant-time CPMG relaxation period [3]. Data were analyzed as described previously to obtain the kinetic parameters of interest, corresponding to a two-site exchange process, $B \underset{k_{EB}}{\overset{k_{BE}}{\rightleftarrows}} E$ [4, 5]. Briefly, a global two-state model (*i.e.* with the same kinetic parameters for all dispersion profiles considered) was fit to the data using the program ChemEx that minimizes the target function $\chi^2 = \sum_i \left(\frac{R^{Exp}_{2,eff,i} - R^{Calc}_{2,eff,i}}{\sigma_i} \right)^2$ by numerically propagating the Bloch-McConnell equations [5, 6]. Here $R^{Exp}_{2,eff,i}$ is the measured effective transverse relaxation rate, $R^{Calc}_{2,eff,i}$ is the calculated value, σ_i is the uncertainty in the measured $R^{Exp}_{2,eff,i}$ rates and the summation extends over all the dispersions of interest. Errors in the fitted parameters were obtained by a covariance matrix analysis [7]. Between 8 and 21 residues were analyzed at each of the 5 temperatures for which CPMG RD were recorded (1, 5.3, 10.4, 15.4 and 20.4 ^{o}C).

Arrhenius analysis of the temperature-dependent rate constants k_{BE} and k_{EB}. The ten extracted k_{BE} and k_{EB} rates (one set of values at each of the 5 temperatures at which RD CPMG profiles were measured; note rates at 15.4 and 20.4 ^{o}C were obtained previously [8]) were fit to the simplest model that describes the temperature dependence of the rates (Fig. S1). Here we have chosen the Arrhenius model to fit the data so that $k_{BE} = A^s e^{\frac{-\Delta H^*}{RT}}$ and $k_{EB} = A^s e^{\frac{-(\Delta H^* - \Delta H + T\Delta S)}{RT}}$ where the constant A^s is given by the relation $A^s = A e^{\frac{\Delta S^*}{R}}$ and the fitting parameters are the changes in enthalpy, entropy and activation enthalpy,

ΔH, ΔS, ΔH^*, respectively, and A^s (see Fig. S1). Best-fit parameters were obtained by nonlinear least squares minimization of a χ^2 target function that is based on the differences between measured and calculated exchange rates. The thermodynamic parameters so obtained were then used to predict the exchange rates at 37 and 50 °C (Fig. S2, Table 1). Uncertainties in the fitted parameters were estimated using a Monte Carlo procedure [7] whereby one hundred synthetic data sets were generated by adding Gaussian random errors based on the experimental uncertainties in the measured rates to the best-fit estimates of k_{BE} and k_{EB} at all five temperatures. Each of these data sets was then fit to extract the thermodynamic parameters listed above, as with the experimental data. The standard deviation in each of the best-fit parameters (based on the 100 trials) was taken as the error in that parameter.

Details of the MD simulations. All MD simulations were performed using the GROMACS 4.5.5 package [9] with the CHARMM27 force field that included the CMAP term [10-12]. Water molecules were represented by the TIP3P potential [13]. Structures from the MD simulations were visualized using the programs VMD [14] and CHIMERA [15]. The initial model for the E state of T4Ltm (*i.e.*, T4L L99A/G113A/R119P) was based on the X-ray structure of the E state of T4L L99A (RCSB id: 3DMV) [16] with G113 and R119 mutated to Ala and Pro, respectively, using the CHIMERA [15] program. This process generated two preliminary structures, one for each rotamer of proline 119, *i.e.*, with the five membered ring in the up or down pucker. The protein molecule was then stripped of all HETATM records in the pdb file and the naked protein was solvated in a dodecahedron box of size 78.9 Å. The protein was subsequently neutralized by the addition of Na^+ cations and an excess of Cl^- anions such that the concentration was ~75 mM in NaCl and the system, now comprising 34,795 atoms, had a net charge of zero. The potential energy of the system was minimized for 50,000 steps using the steepest decent method followed first by 50 ps of NVT MD simulation at 310 K and then 10.05 ns of NPT MD simulation at 310K, 1 Bar. During the NVT and NPT runs the backbone atoms of residues 100 to 120 were restrained to their starting positions with a 400 kJ/mol.nm^2 force constant followed by a 10 ns NPT run with no restraints. During a 50 ns NPT MD run that followed, the coordinates of all the atoms in the system were stored every 10 ns to generate 5 starting structures. The procedure was carried out for each of the two preliminary structures, leading to a total of 10 starting structures. Pro119 routinely interconverted between the two puckers during the subsequent MD simulations and hence the results did not depend on the starting structures used.

Eight long production NPT runs (37 °C) were started from each of these 10 structures and protein coordinates were stored every 25 ps for further analysis, with the coordinates of the entire system saved every 2 ns. The length of these eighty trajectories varied from 840 ns to 1.3 μs, leading to a total run

time of ~83 μs. Ten E to B transitions were observed in these eighty simulations. A total of 184 structures were extracted during intervals when the molecules where making the E to B transition in these ten trajectories. Three short 100 ns runs were started from each of these 184 structures leading to an additional ~55 μs of simulation, for a total time of ~138 μs. A similar protocol was used to generate the 50 °C MD data, with forty ~1 μs long trajectories and four hundred and forty short 100 ns trajectories obtained for a total time of ~90 μs.

In all of the simulations, van der Waals and electrostatic terms were applied with a cutoff of 9 Å. Long-range electrostatics were treated using the particle mesh Ewald (PME) method [17] and bond lengths constrained using the LINCS method [18], with hydrogens treated as virtual atoms using the vsite option in GROMACS [9, 19]. A leapfrog integrator with a timestep of 4 fs was used to integrate Newton's equations of motion. The temperature of the simulation was controlled by using the velocity rescaling method of Bussi et. al [20] while the pressure was kept constant with the Parrinello-Rahman barostat [21]. All the simulations were performed on the GPC cluster of the SCINET supercomputing facility [22]. Each simulation made use of four quadcore processors spread over two nodes, leading to a computational speed of 40-45 ns/day.

In Rosetta calculations of the RD CPMG-derived NMR model of the B state most of the residues in the protein were constrained to be identical to the crystal structure of T4L L99A while regions comprising residues with changes in chemical shifts between ground and excited states were allowed to vary structurally, as described previously [23, 24]. We choose not to use the resultant RD-CPMG derived structure as representing the B state in this work because of the potential bias that may be introduced by fixing the majority of the protein structure to be identical in E and B states, although this is most certainly consistent with the CPMG data. Instead a structure representing the B state was generated from MD simulations. This was accomplished by taking different B state structures obtained from the long trajectories (starting from the E conformer) that made the transition to B, pooling them together and clustered them using an RMSD metric that included backbone N, Cα, carbonyl C, and O atoms of residues 3 to 155 and all the heavy atoms of Phe114. The central structure of the largest cluster was chosen to be the representative structure of the B state (Atomic coordinates of this B state structure are provided in Supporting Material). The backbone RMSD between this structure and the RD NMR-derived model of the B state is 1.9 Å. The two structures are similar, Fig. S3, and the MD derived structure is in complete agreement with the NMR RD data. The main differences localize to the loop between helices E and F. Loop residues Gln105 and Met 106 were placed in the left handed helical region or in the β sheet region of the Ramachandran plot in the RD-NMR derived structures but are in

the right handed helical region in the MD derived structure of the B state. The φ/ψ values of Gln 105 and Met 106 could not be unambiguously determined using standard programs such as TALOS+ [25] that were available to us at the time of the structure calculations in 2010/11. TALOS+ did suggest that these residues could be in the left-handed helical region but could not unambiguously make any prediction. However the newer improved TALOS-N program [26, 27] unambiguously predicts that Gln105 and Met106 are in the right-handed helical region, consistent with the MD derived structure.

Approximate transition path times (TPTs) were estimated from RMSD *vs.* time graphs (like those in Fig. 2 A, B) for the ten (17) long trajectories computed at 37 ^{o}C (50 ^{o}C) for which an E to B transition was observed (Fig. S9). Molecules were considered to have left the E state if the RMSD to the E state crystal structure (T4 L99A 3DMV) was greater than 0.7 Å and were determined to have entered the B state if the RMSD to the B state reference structure (see above) was less than 0.9 Å. The backbone N, Cα, C and O atoms of residues 100 to 120 and all heavy atoms in Phe114 and Leu133 were used for the RMSD calculations.

MSM Analysis of the MD trajectories. The input data for the MSM analysis consisted of ~184 μs of simulation (37 ^{o}C) comprising 80 long and 552 shorter trajectories, as described above. We have used structures from these trajectories that were obtained every 100 ps. The first 50 ns and 5 ns of each of the long and short trajectories, respectively, was discarded, leading to a total of 1,315,000 structures for analysis. Using a combination of RMSD and dihedral angle metrics, a two-hundred-microstate MSM was generated. Atoms used in the RMSD clustering included the backbone Cα atoms of residues 78 - 155, the backbone N, C and O atoms of residues 100 - 120 and all the heavy atoms of Phe114 and Leu133. The angles included in the dihedral component of the clustering were φ and ψ of Gly110 and Phe114 and χ_1 of Phe114 and Leu133. The hybrid k-centers/k-medoids protocol was used in a two-step manner as recommended [28]. First 200 cluster centers were generated using only 10% of the data (subsampled at every 1 ns instead of 0.1 ns). All the remaining structures were then assigned to each of these 200 cluster centers. The implied timescales calculated from this 200 state model (Fig. S4) flatten at 20 ns and a transition probability matrix was constructed at a lag time of 22.5 ns. Similar implied timescales were obtained when the clustering and MSM analysis were carried out with different number of microstates (Fig S4).

A simpler MSM was constructed by lumping the 200 microstates into 19 macrostates using the PCCA+ procedure [29]. The procedure was performed at different lag-times for different numbers of macrostates and the 19-macrostate model chosen because the implied timescales of this model followed those [30] of the 200 microstate model (Fig. S4). Further, the transition probability matrix, $T(\tau)$,

constructed at a lag-time of τ = 22.5 ns satisfies the Chapman-Kolmogorov test (Fig. S5A) [31]. As described in the main text, rates and populations for the B-E transition, assuming a two-state model, were obtained from the eigenvalues and eigenvectors of $T(\tau)$. Uncertainties in these estimated parameters were generated using a bootstrap procedure [7] whereby 25 new sets of input data for calculating MSMs where obtained by randomly choosing trajectories from the 80 long and 550 short trajectories with repetition. The standard deviation in each of the parameters calculated from the 25 sets was assumed to be the uncertainty in the parameter. Data analysis of the simulations performed at 50 ^{o}C followed the same procedure as for 37 ^{o}C, described above (Fig. S4, S5B).

Free energy surfaces, $f(x,y)$, (Figs. 4, S7) were calculated according to the relation $f(x,y)/k_B T = \ln\left(\sum_{i=0}^{19} \pi_i h_i(x,y)\right)$ [32]. Here x, y are the parameters of interest, π_i is the population of state i obtained from the first eigenvector of the transition probability matrix [33] and $h_i(x,y)$ is the normalized histogram of state i.

T4L L99A MD simulations: Initial simulations, at temperatures ranging from 37 to 227 ^{o}C, were performed starting from the published crystal structure coordinates (3DMV [16]) of the E state. An E to B transition was observed in a trajectory simulated at 167 ^{o}C. Structures from the transition region were chosen as initial coordinates for 500 short 100 ns MD runs at 37 ^{o}C. The endpoint structures were analyzed and were found to adopt not only conformers corresponding to states B and E but also structures similar to those observed during the E to B trajectory of T4Ltm (Fig. 6).

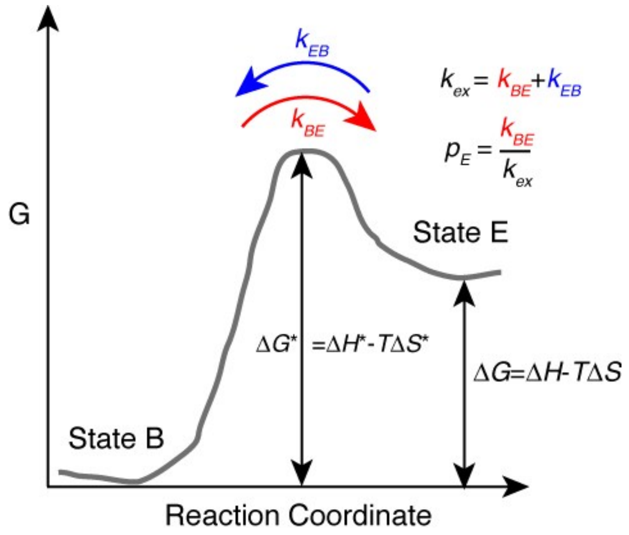

Figure S1. Schematic diagram of the energy landscape for the exchange between states B and E, assuming two-state interconversion. The kinetic and thermodynamic parameters are as indicated. The major state B is the reference state for the calculation of all thermodynamic parameters (Table 2).

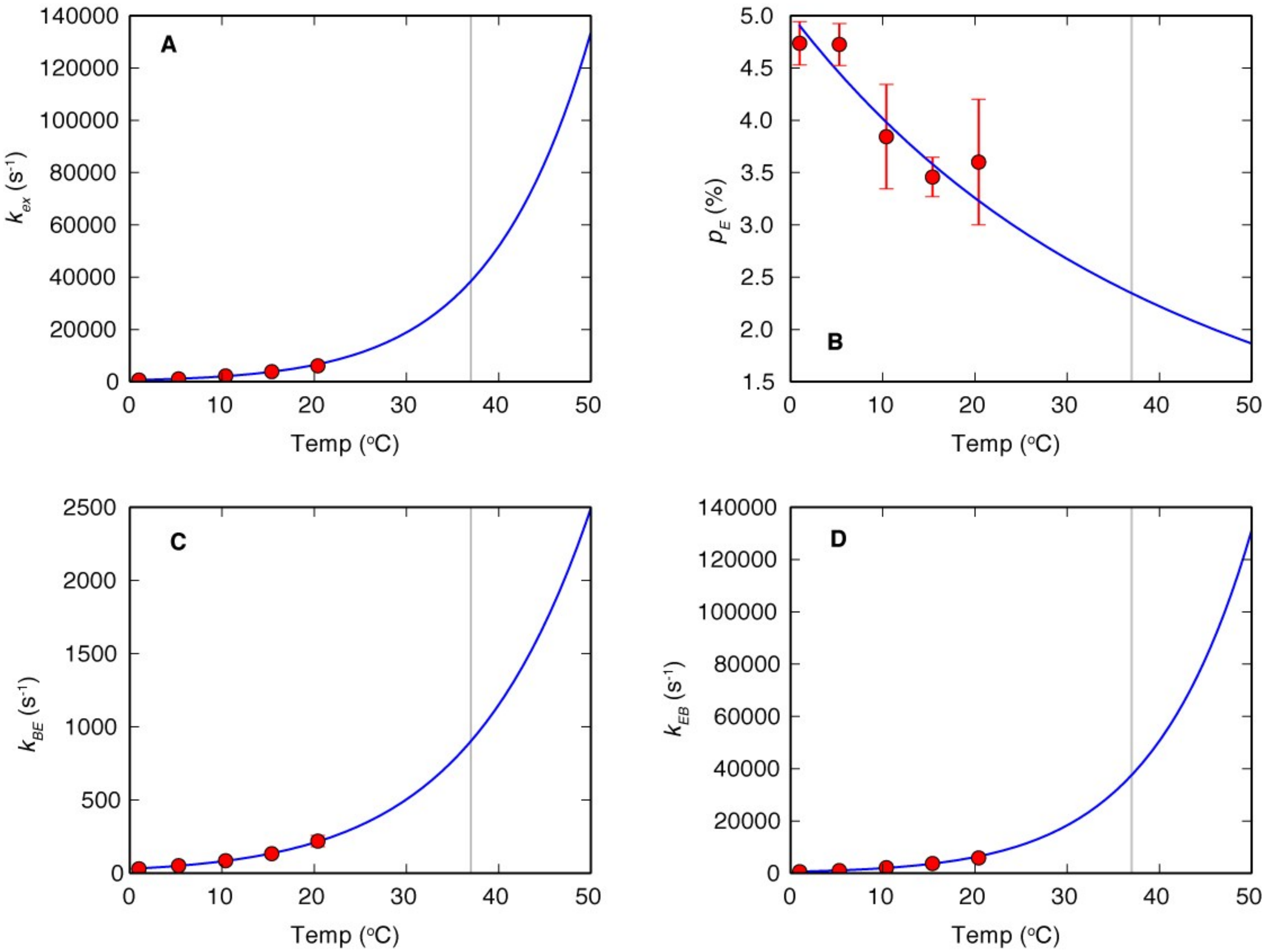

Figure S2. Variation of rates, $k_{ex} = k_{EB}+k_{BE}$, k_{BE}, k_{EB} (A, C, D) and the fractional population of E, p_E, (B) with temperature based on CPMG RD data. The strong temperature dependence of the B/E interconversion and p_E is clear. The blue lines in A-D are derived from fits of the temperature dependent rates k_{EB}, k_{BE} using the Arrhenius model described in SI Materials and Methods, with error bars shown in red when they are larger than the sizes of the circles that denote experimental data. The vertical grey line is drawn at 37 °C.

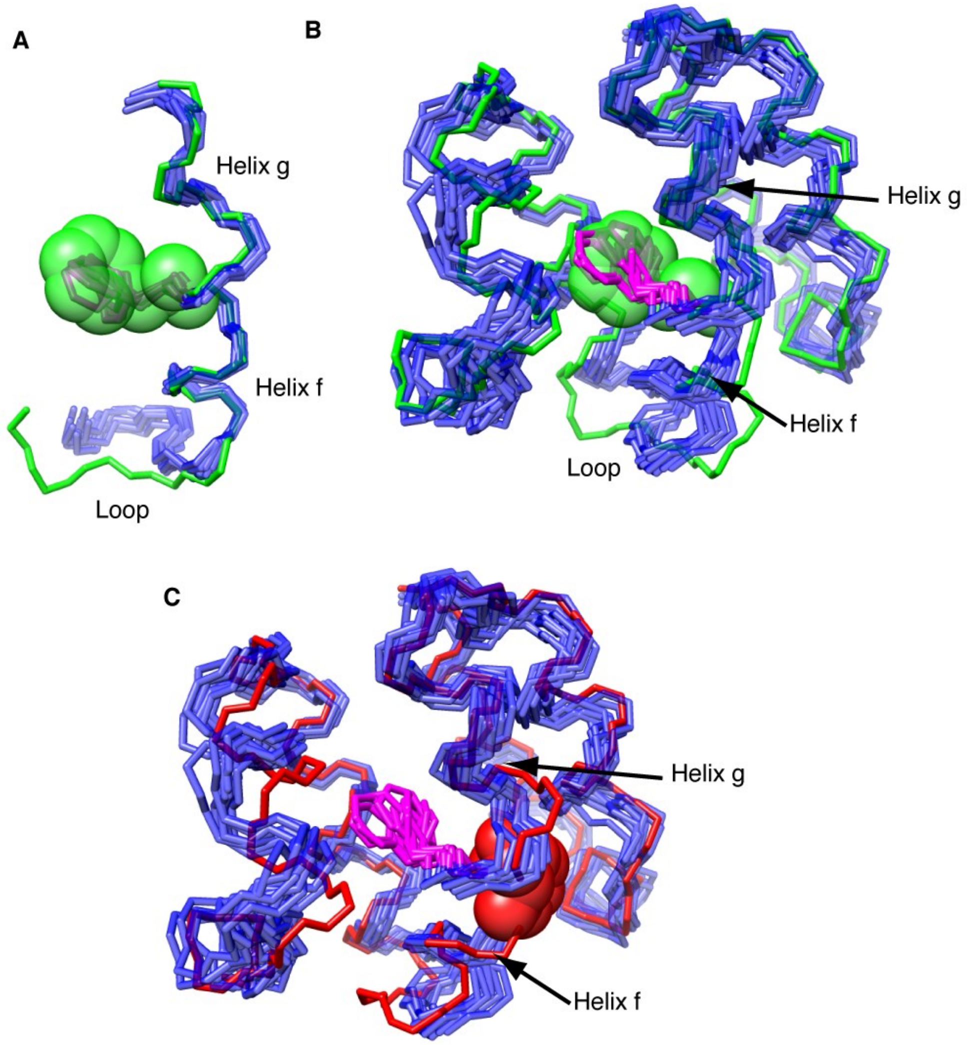

Figure S3. The MD and CPMG RD NMR derived structures of the B state superimpose well. A) The backbone conformation of residues in helices f and g are very similar in the MD (blue, atomic coordinates are provided in Supporting Material) and CPMG RD (green, PDB code: 2LCB) derived structures of the B state. The backbone N, Cα and C atoms of residues 104 to 120 are shown, with Phe114 in CPK representation for the RD CPMG derived structure and in magenta for the ten randomly chosen MD structures of the B state. The superposition is based on the backbone atoms of residues 108 to 120 showing that they are nearly identical, with a difference only in the conformation of the loop that connects helices e and f (See SI, Details of the MD simulations). B) A superposition of the backbone atoms of residues 77 to 155 shows that the overall conformations of the MD and RD CPMG NMR derived structures are very similar despite differences in the loop structure. The Phe114 sidechains from the different structures overlap well with each other and in all cases are localized to the cavity. C) In contrast, the crystal structure of the E state (red) is clearly different from the MD derived B state structures with a kink between helices f and g that exposes Phe114 to the solvent. In the crystal structure the sidechain of Phe114 is shown as a CPK model.

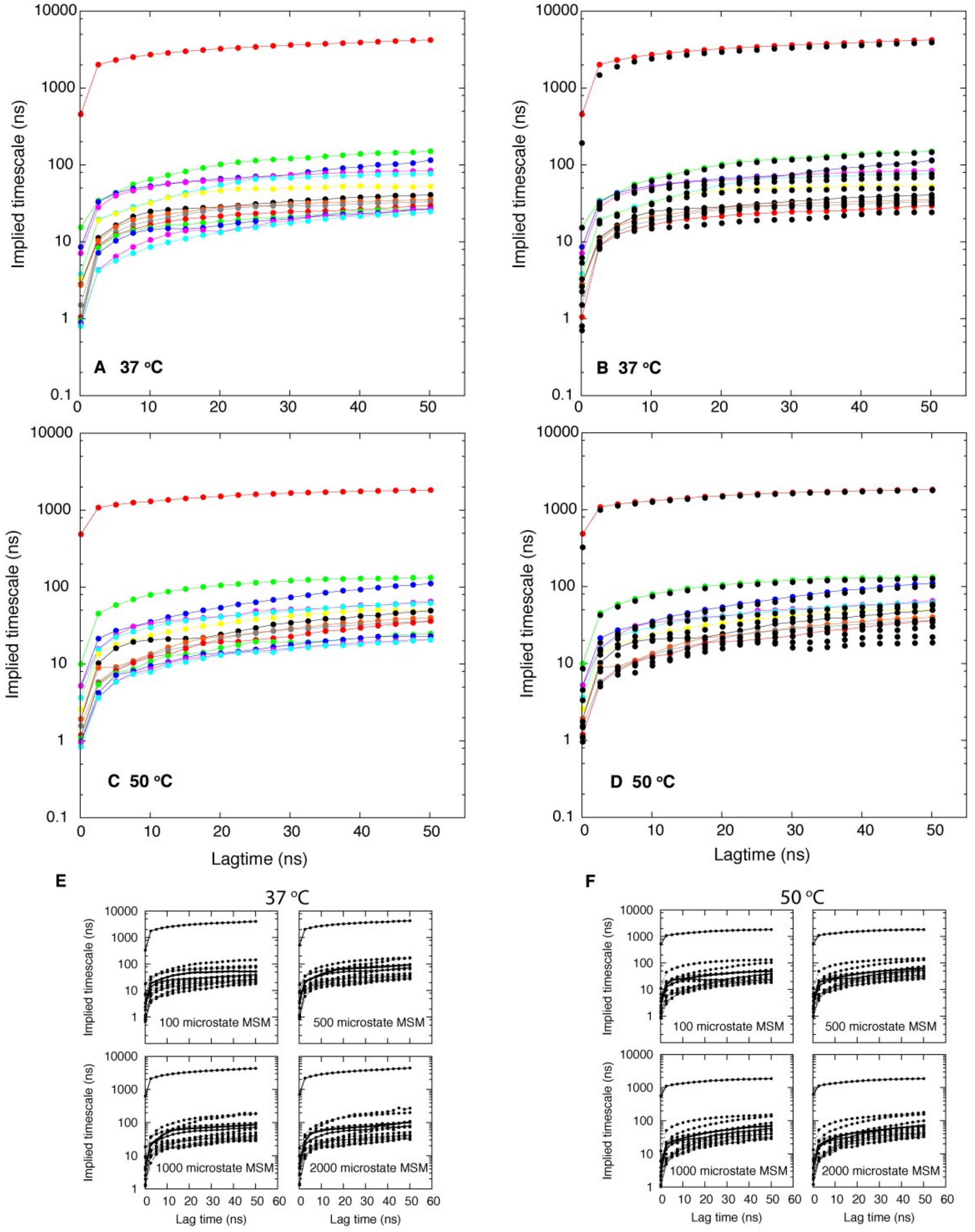

Figure S4. Implied timescales plateau by approximately 20 ns in the MSM analysis of the MD simulations at 37 °C and 50 °C. A,C) The slowest fifteen implied timescales for the 200 microstate model at 37 and 50 °C. Timescales were calculated from the eigenvalues of the transition probability matrix, as described in the text and in reference [28]. Because the profiles are essentially constant by 20 ns, a delay of 22.5 ns was used for all the analyses. B, D) The ten slowest implied timescales of the 19 macrostate model shown as black dots follow the implied timescales of the 200 microstate model (superimposed in color). E, F) Very similar implied timescales are obtained when different numbers of microstates are used, supporting the use of the 200 microstate MSM model.

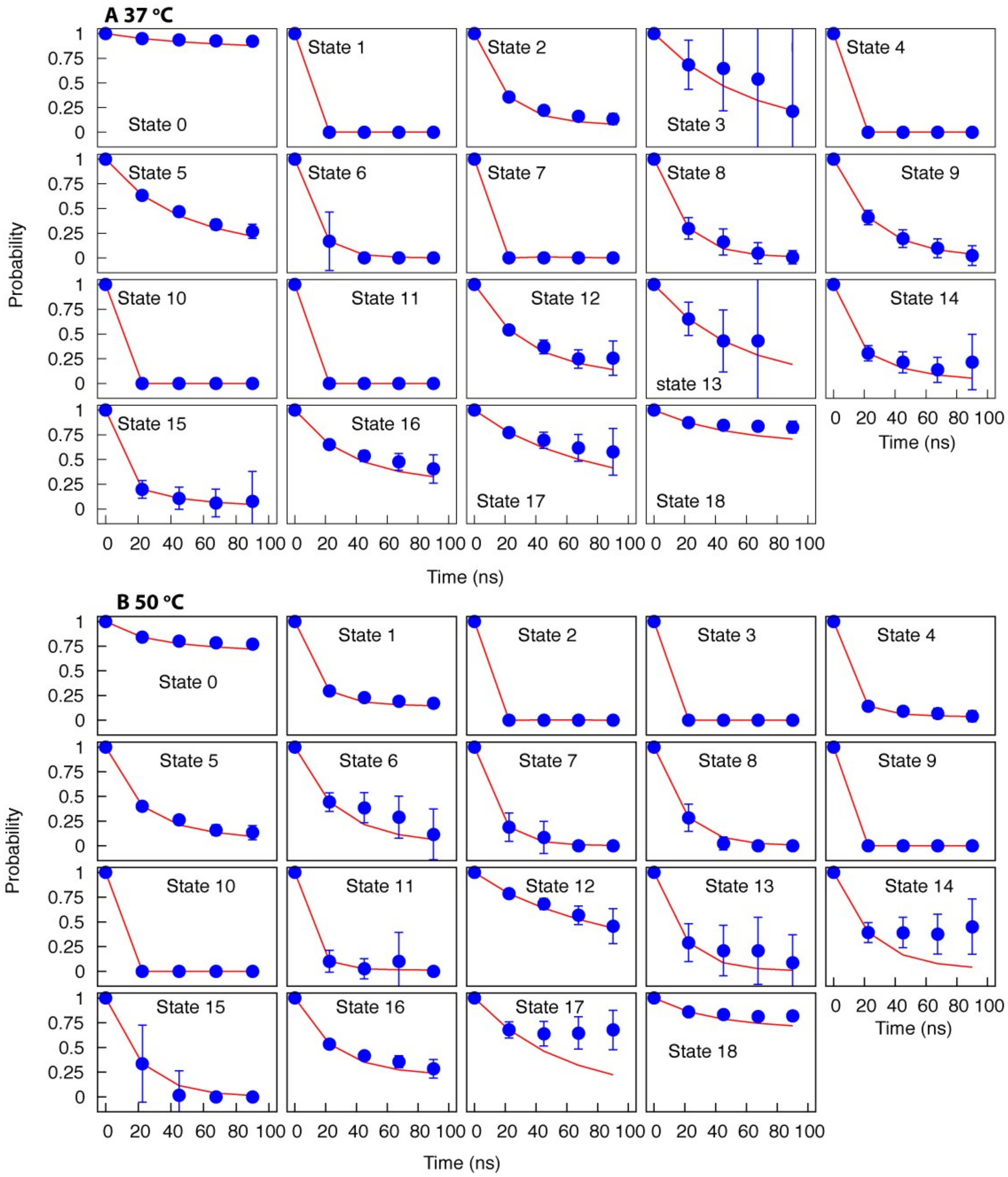

Figure S5. Chapman–Kolmogorov test [31] validating the 19 state MSMs constructed at 37 °C (A) and 50 °C (B). The evolution of the population for each of the 19 states predicted by the 22.5 ns lag-time model (via $P(n\tau) = P(0)T(\tau)^n$, $\tau = 22.5$ ns, where the elements of the population vector $P(0)$ are 1 for the state in question and 0 elsewhere) is shown with the red line, while the blue circles illustrate how the population of each state evolves from direct analysis of the trajectories. Most of the points lie within 1σ of the line.

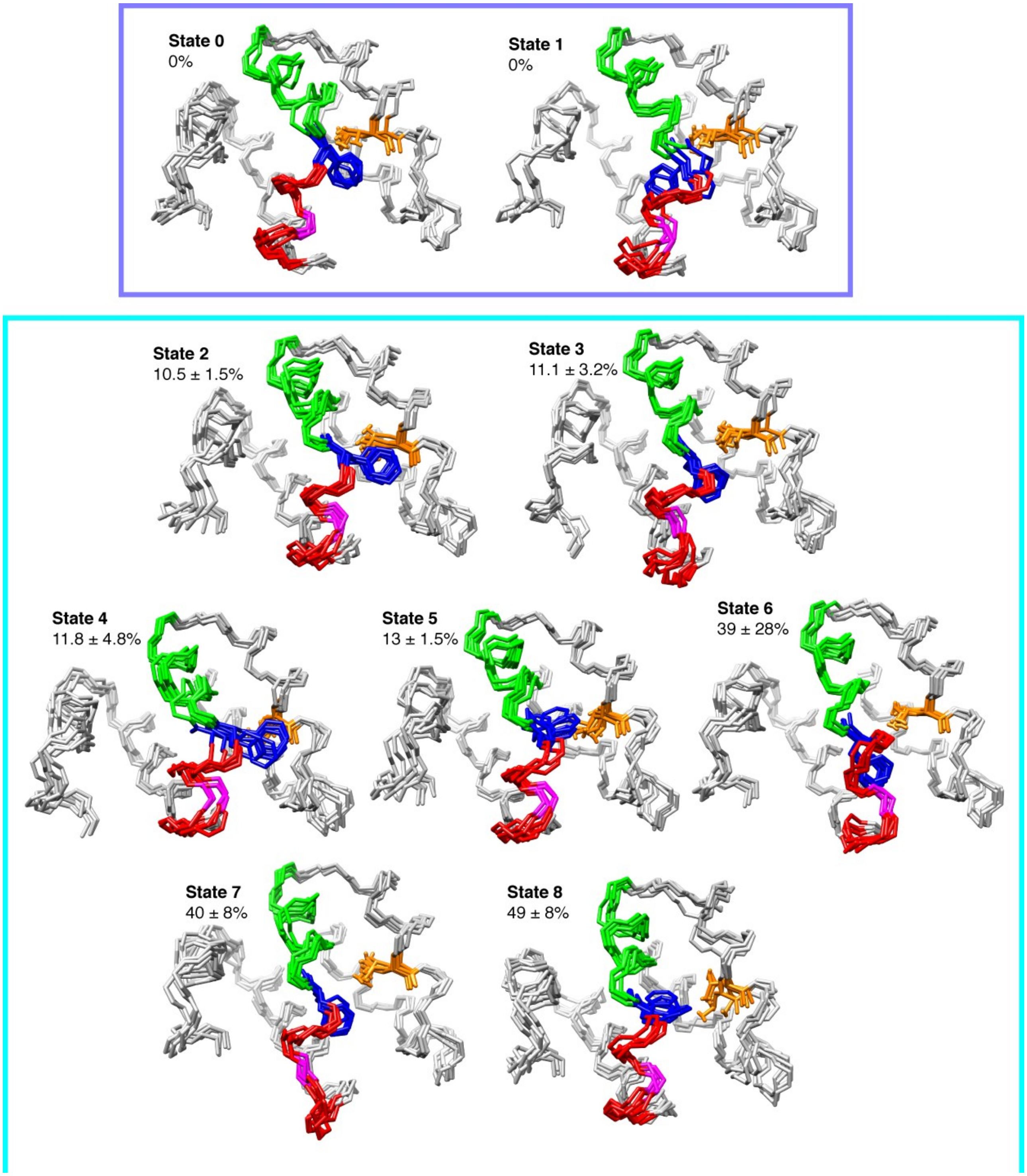

Figure continues on the next page

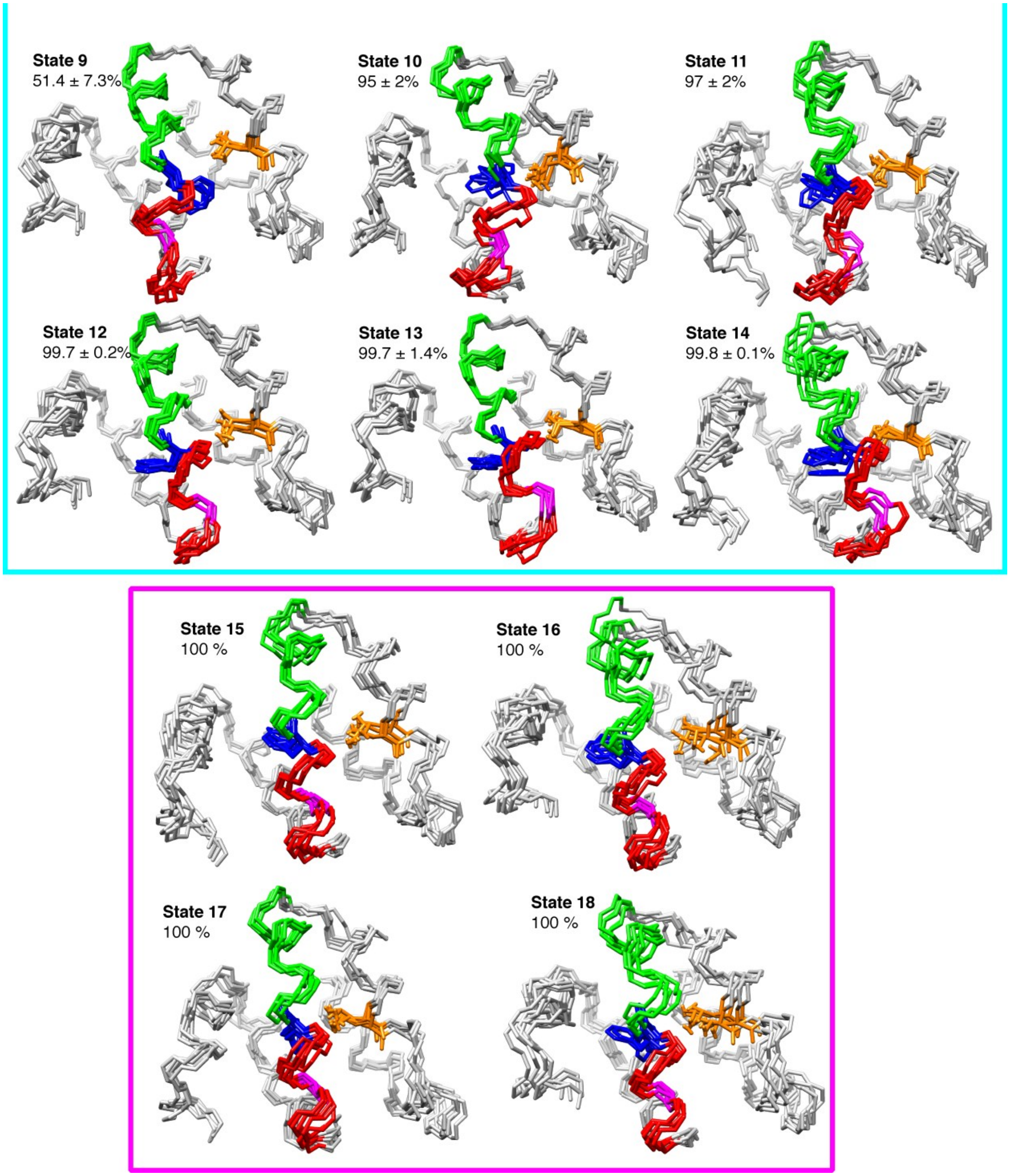

Figure S6. Conformers in the same macrostate are structurally very similar. Five randomly chosen structures from each of the 19 states of the MSM calculated from the 37 ^{o}C MD data are shown. A pdb file containing the atomic coordinates for five conformers from each of the 19 states is available in Supporting Material. Residues 78-155 are shown with helix f in red, helix g in green, Phe114 in blue and Leu133 in orange. Backbone N, Cα, C atoms are indicated along with the sidechains of Phe114 and Leu133. State 0 forms the E state and states 15-18 the B state. State 0 and 1 have been grouped together because 1 only exchanges with 0. Committor values are shown for each state as percentages.

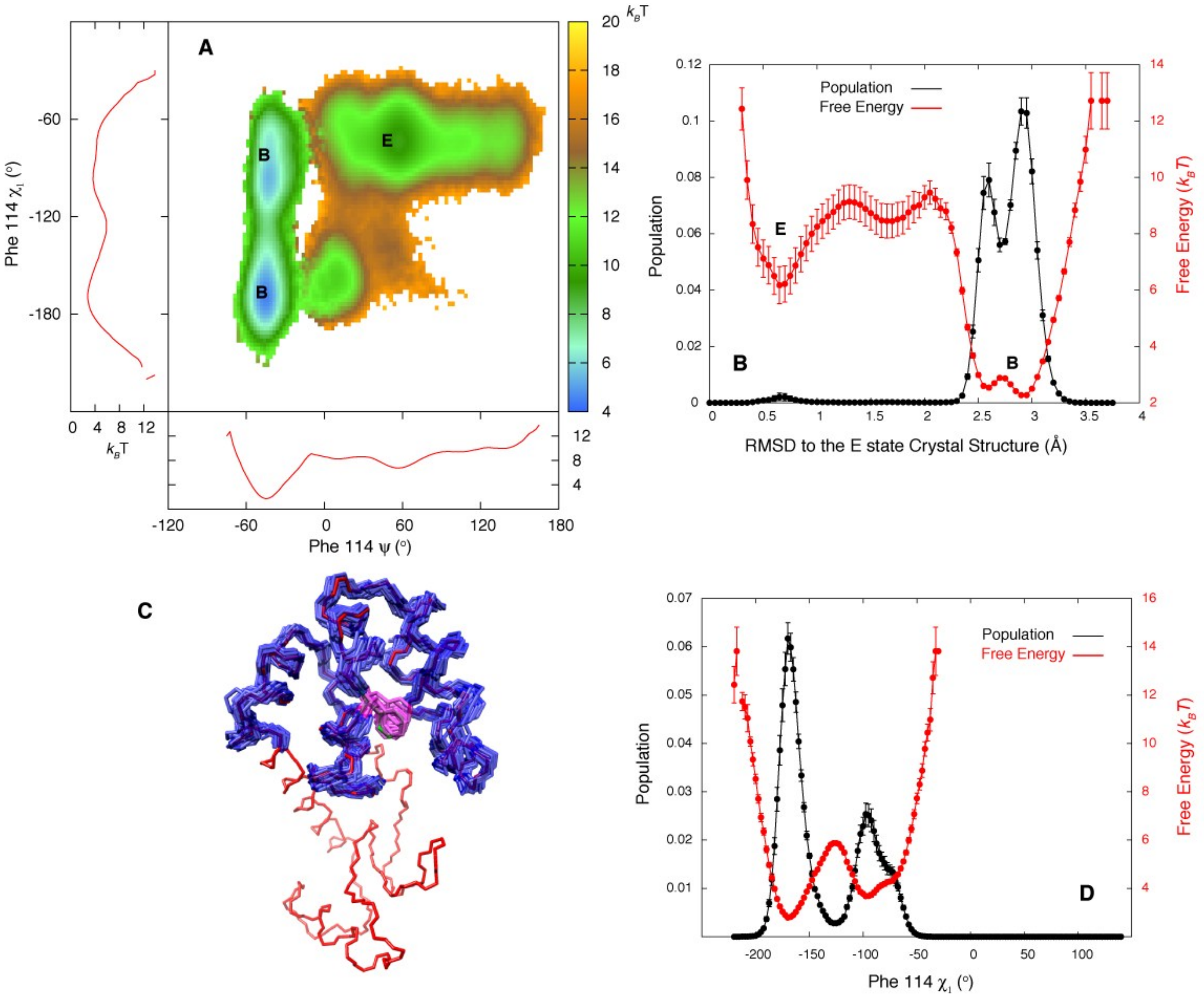

Figure S7. The force field reproduces the free energy surface on which the protein moves in solution.

A) A 2D potential of mean force (PMF) showing the location of the buried and exposed states, 37°C. For Phe114 ψ = ~+50°, χ_1 = ~-60° in state E, as found in the T4L L99A crystal structure [16], and transitions to ψ ~-50°, χ_1 = ~-170° in B as predicted by the RD CPMG NMR derived structure of the B state [24]. B) 1D PMF showing that the E state is very similar to the T4L L99A crystal structure, with an RMSD of ~0.6 Å. C) Backbone atoms of residues 78-155 from 25 randomly chosen structures (blue) from the E state superimposed on the T4L L99A crystal structure (red) establishing the close similarity between the sets of structures. (Atomic coordinates of five randomly chosen E state conformers (state 0) are available in Supporting Material). Backbone atoms of all residues are shown for the crystal structure, while those from residues 78-155 are shown for E from the simulations. Phe114 is shown in all of the structures and is colored green in the crystal structure and magenta in the E form from the simulations. The structures from simulations are partially transparent so that the crystal structure can been seen through them. D) 1D PMF of Phe114 χ_1 establishing that the trans conformation is the major state in B as predicted by the RD CPMG derived structure of the B state. On the basis of the chemical shifts of T4Ltm Phe 114 χ_1 is predicted to populate [26, 27] the trans conformation to ~80% which is in reasonable agreement with the value of ~65% obtained here. The PMF is calculated from all conformations but the contribution from state E is negligible (< 2%). All PMFs were computed from MD data at 37 °C.

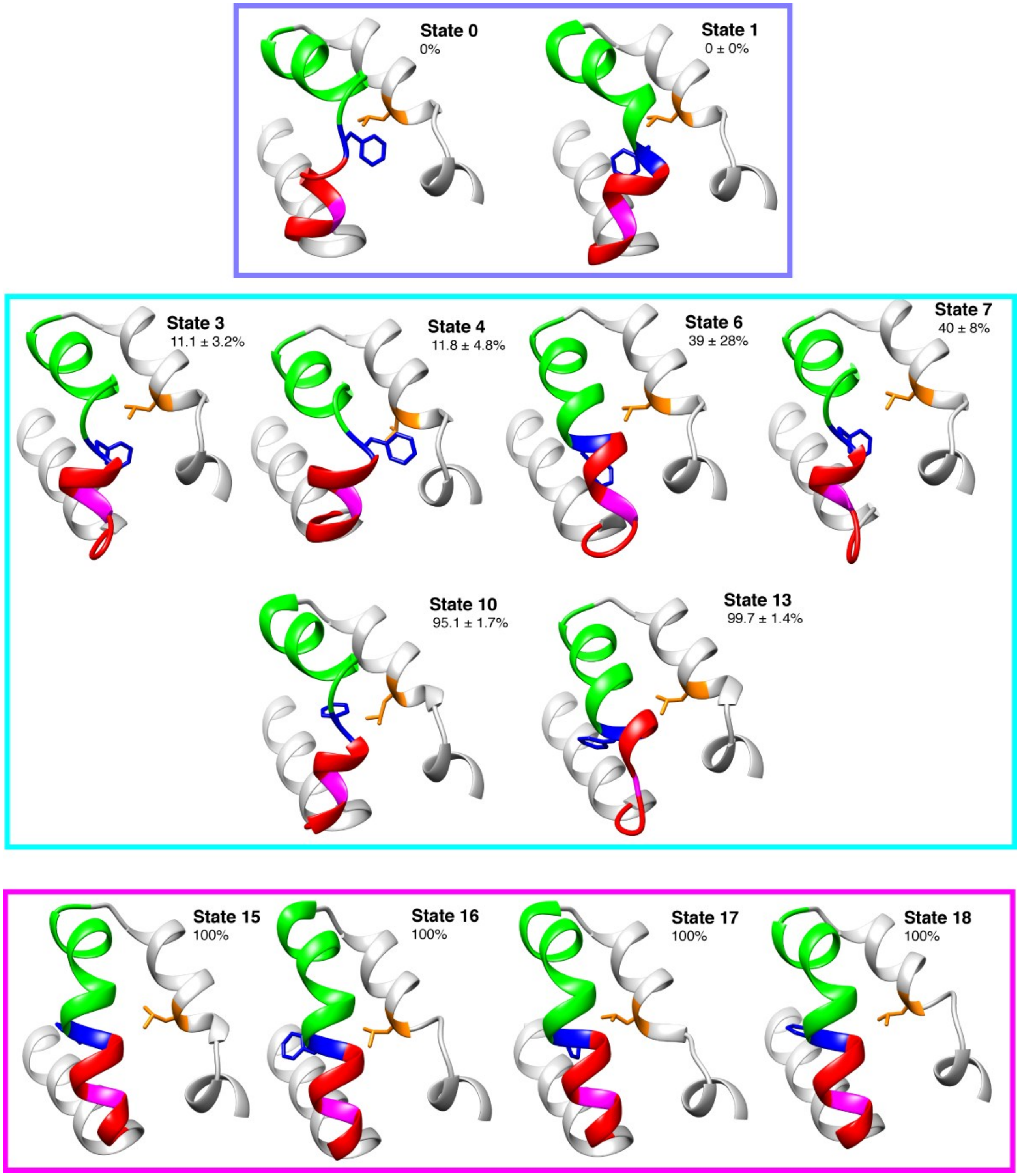

Figure S8. Ribbon representation showing the states that are not indicated in Fig. 5 of the main text. State 0 is the E form, and because state 1 exchanges only with it, states 0 and 1 can be grouped together (although the population of state 1 is much less than that of state 0). States 15-18 are all structurally very similar and comprise state B (Table S1). The remaining states are intermediates. Commitor values for each of the states are shown in percentages. Atomic coordinates of five randomly chosen structures from each of the 19 states are available in Supporting Material.

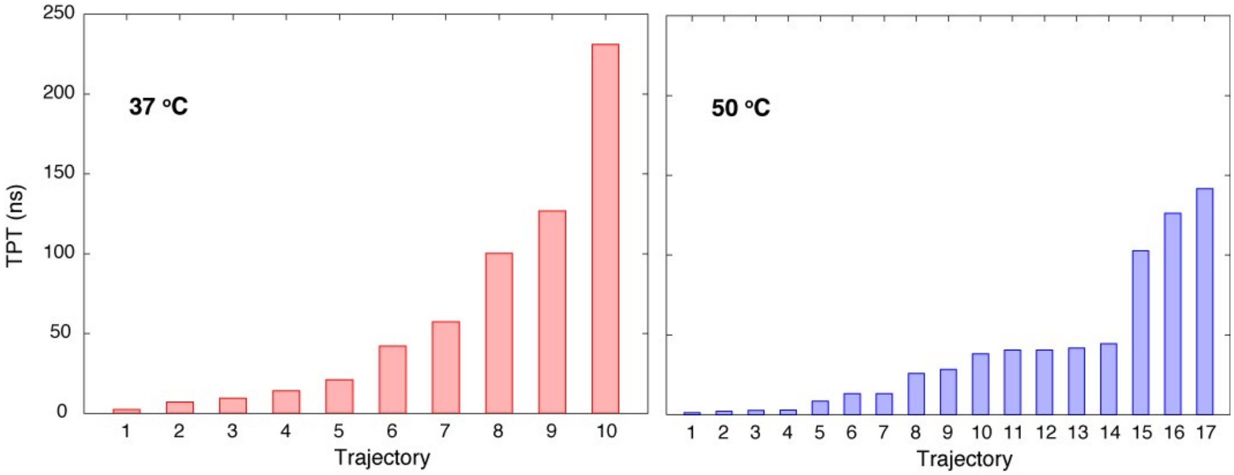

Figure S9. The transition path times (TPTs) vary by two orders of magnitude. The TPTs observed in the MD trajectories at 37 ^{o}C (left) and 50 ^{o}C (right), sorted in ascending order.

Table S1. Populations and committor values of the 19 states as derived from the MSM describing the E-B interconversion of T4Ltm and 37 ^{o}C (A) and 50 ^{o}C (B).

A.

State	Population(%)	Error(%)	Committor(%)	Error(%)
0	1.34e+00	1.09e+00	0.0	0.0
1	1.32e-04	2.01e-04	0.0	0.0
2	1.03e-01	8.47e-02	10.5	1.4
3	8.41e-04	1.64e-03	11.1	3.2
4	2.56e-04	2.53e-04	11.8	4.8
5	1.07e-01	9.37e-02	13.0	1.5
6	3.64e-04	6.33e-04	38.5	27.6
7	1.36e-04	1.24e-04	39.7	8.3
8	7.25e-03	5.96e-03	48.7	8.1
9	6.84e-03	5.01e-03	51.4	7.3
10	1.55e-03	1.27e-03	95.1	1.7
11	9.49e-03	2.99e-03	97.0	2.1
12	3.82e+00	1.06e+00	99.7	0.2
13	3.54e-01	2.27e-01	99.7	0.4
14	8.74e-01	3.11e-01	99.8	0.1
15	7.56e-01	2.04e-01	99.9	0.1
16	2.34e+01	2.09e+00	99.9	0.1
17	7.25e+00	1.68e+00	99.9	0.1
18	6.20e+01	4.14e+00	100.0	0.0

B.

State	Population(%)	Error(%)	Committor(%)	Error(%)
0	5.35e-01	2.06e-01	0.0	0.0
1	1.17e-01	4.35e-02	6.6	0.9
2	5.46e-04	2.67e-04	7.0	0.9
3	2.74e-04	3.55e-04	13.3	33.6
4	3.14e-02	1.01e-02	15.6	1.1
5	5.65e-02	1.94e-02	22.3	3.7
6	8.83e-03	3.13e-03	24.8	6.2
7	2.77e-03	1.21e-03	33.2	7.7
8	2.77e-03	5.15e-04	57.5	24.1
9	2.61e-03	1.42e-03	95.8	0.4
10	1.76e-03	7.36e-04	96.4	0.7
11	3.95e-01	1.29e-01	99.7	0.1
12	8.33e+00	1.96e+00	99.8	0.1
13	2.76e-01	1.44e-01	99.8	0.1
14	1.42e+00	5.81e-01	99.9	0.1
15	8.93e-02	5.44e-02	99.9	0.1
16	2.09e+01	2.93e+00	99.9	0.0
17	3.05e+00	1.81e+00	100.0	0.0
18	6.48e+01	3.88e+00	100.0	0.0

Supporting Atomic Coordinates

The heavy atom coordinates of the MD derived reference B state structure are available in Supporting Material (B_state_from_md.pdb). A pdb file (five_structures_from_each_state.pdb) containing heavy atom coordinates of five randomly chosen conformers from each of the 19 states is available in Supporting Material. The structures are ordered according to the state to which they belong. The first five structures belong to state 0, the next five to state 1 and so on.

Supporting Movies

Two movies (Trajectory-1_530-560ns.avi & Trajectory-2_490-610ns.avi) showing the E to B transition in Trajectories 1 & 2 (Figure 2) are included in Supporting Material. The length of each of the trajectories is the same as in Figure 2 and the coloring scheme is similar to Figure 5.

References

1. F. Delaglio, S. Grzesiek, G. W. Vuister, G. Zhu, J. Pfeifer and A. Bax, *J Biomol NMR*, 1995, **6**, 277-293.
2. T. D. Goddard and D. G. Kneller, *SPARKY 3 University of California, San Francisco*.
3. F. A. Mulder, N. R. Skrynnikov, B. Hon, F. W. Dahlquist and L. E. Kay, *J Am Chem Soc*, 2001, **123**, 967-975.
4. F. A. Mulder, A. Mittermaier, B. Hon, F. W. Dahlquist and L. E. Kay, *Nat Struct Biol*, 2001, **8**, 932-935.
5. D. M. Korzhnev, X. Salvatella, M. Vendruscolo, A. A. Di Nardo, A. R. Davidson, C. M. Dobson and L. E. Kay, *Nature*, 2004, **430**, 586-590.
6. H. M. McConnell, *J Chem Phys*, 1958, **28**, 430-431.
7. W. H. Press, B. P. Flannery, S. A. Teukolsky and W. T. Vetterling, *Numerical Recipes in C. The Art of Scientific Computing* Cambridge University Press, Cambridge (UK), Second Edition edn., 1992.
8. P. Vallurupalli, G. Bouvignies and L. E. Kay, *J Phys Chem B*, 2011, **115**, 14891-14900.
9. D. Van der Spoel, E. Lindahl, B. Hess, G. Groenhof, A. E. Mark and H. J. C. Berendsen, *Journal of Computational Chemistry*, 2005, **26**, 1701-1718.
10. P. Bjelkmar, P. Larsson, M. A. Cuendet, B. Hess and E. Lindahl, *J Chem Theory Comput*, 2010, **6**, 459-466.
11. A. D. Mackerell, M. Feig and C. L. Brooks, *Journal of Computational Chemistry*, 2004, **25**, 1400-1415.
12. A. D. MacKerell, D. Bashford, M. Bellott, R. L. Dunbrack, J. D. Evanseck, M. J. Field, S. Fischer, J. Gao, H. Guo, S. Ha, D. Joseph-McCarthy, L. Kuchnir, K. Kuczera, F. T. K. Lau, C. Mattos, S. Michnick, T. Ngo, D. T. Nguyen, B. Prodhom, W. E. Reiher, B. Roux, M. Schlenkrich, J. C. Smith, R. Stote, J. Straub, M. Watanabe, J. Wiorkiewicz-Kuczera, D. Yin and M. Karplus, *J Phys Chem B*, 1998, **102**, 3586-3616.
13. W. L. Jorgensen, J. Chandrasekhar, J. D. Madura, R. W. Impey and M. L. Klein, *Journal of Chemical Physics*, 1983, **79**, 926-935.
14. W. Humphrey, A. Dalke and K. Schulten, *J Mol Graph Model*, 1996, **14**, 33-38.
15. E. F. Pettersen, T. D. Goddard, C. C. Huang, G. S. Couch, D. M. Greenblatt, E. C. Meng and T. E. Ferrin, *J Comput Chem*, 2004, **25**, 1605-1612.
16. L. J. Liu, W. A. Baase and B. W. Matthews, *Journal of Molecular Biology*, 2009, **385**, 595-605.
17. T. Darden, D. York and L. Pedersen, *Journal of Chemical Physics*, 1993, **98**, 10089-10092.

18. B. Hess, H. Bekker, H. J. C. Berendsen and J. G. E. M. Fraaije, *Journal of Computational Chemistry*, 1997, **18**, 1463-1472.
19. K. A. Feenstra, B. Hess and H. J. C. Berendsen, *Journal of Computational Chemistry*, 1999, **20**, 786-798.
20. G. Bussi, D. Donadio and M. Parrinello, *Journal of Chemical Physics*, 2007, **126**.
21. M. Parrinello and A. Rahman, *Journal of Applied Physics*, 1981, **52**, 7182-7190.
22. C. Loken, D. Gruner, L. Groer, R. Peltier, N. Bunn, M. Craig, T. Henriques, J. Dempsey, C. H. Yu, J. Chen, L. J. Dursi, J. Chong, S. Northrup, J. Pinto, N. Knecht and R. V. Zon, *Journal of Physics: Conference Series*, 2010, **256**.
23. P. Vallurupalli, D. F. Hansen and L. E. Kay, *Proc Natl Acad Sci U S A*, 2008, **105**, 11766-11771.
24. G. Bouvignies, P. Vallurupalli, D. F. Hansen, B. E. Correia, O. Lange, A. Bah, R. M. Vernon, F. W. Dahlquist, D. Baker and L. E. Kay, *Nature*, 2011, **477**, 111-114.
25. Y. Shen, F. Delaglio, G. Cornilescu and A. Bax, *J Biomol NMR*, 2009, **44**, 213-223.
26. Y. Shen and A. Bax, *J Biomol NMR*, 2013, **56**, 227-241.
27. Y. Shen and A. Bax, *Methods Mol Biol*, 2015, **1260**, 17-32.
28. K. A. Beauchamp, G. R. Bowman, T. J. Lane, L. Maibaum, I. S. Haque and V. S. Pande, *J Chem Theory Comput*, 2011, **7**, 3412-3419.
29. P. Deuflhard and M. Weber, *Linear Algebra and its Applications*, 2005, **398**, 161-184.
30. G. R. Bowman, in *Protein Dynamics*, ed. D. R. Livesay, Humana Press, 2014, ch. 8.
31. J. H. Prinz, H. Wu, M. Sarich, B. Keller, M. Senne, M. Held, J. D. Chodera, C. Schutte and F. Noe, *J Chem Phys*, 2011, **134**, 174105.
32. D. Shukla, Y. L. Meng, B. Roux and V. S. Pande, *Nat Commun*, 2014, **5**, 1-11.
33. F. Noe and S. Fischer, *Curr Opin Struct Biol*, 2008, **18**, 154-162.
